# Whole exome sequencing reveals *HSPA1L* as a genetic risk factor for spontaneous preterm birth

**DOI:** 10.1371/journal.pgen.1007394

**Published:** 2018-07-12

**Authors:** Johanna M. Huusko, Minna K. Karjalainen, Britney E. Graham, Ge Zhang, Emily G. Farrow, Neil A. Miller, Bo Jacobsson, Haley R. Eidem, Jeffrey C. Murray, Bruce Bedell, Patrick Breheny, Noah W. Brown, Frans L. Bødker, Nadia K. Litterman, Pan-Pan Jiang, Laura Russell, David A. Hinds, Youna Hu, the 23andMe Research Team, Antonis Rokas, Kari Teramo, Kaare Christensen, Scott M. Williams, Mika Rämet, Stephen F. Kingsmore, Kelli K. Ryckman, Mikko Hallman, Louis J. Muglia

**Affiliations:** 1 PEDEGO Research Unit and Medical Research Center Oulu, University of Oulu and Department of Children and Adolescents, Oulu University Hospital, Oulu, Finland; 2 Division of Human Genetics, Center for Prevention of Preterm Birth, Perinatal Institute, Cincinnati Children’s Hospital Medical Center, Department of Pediatrics, University of Cincinnati College of Medicine, March of Dimes Prematurity Research Center Ohio Collaborative, Cincinnati, Ohio, United States of America; 3 Department of Population and Quantitative Health Sciences, Case Western Reserve University School of Medicine, Cleveland, Ohio, United States of America; 4 Center for Pediatric Genomic Medicine, Children's Mercy Hospital, Kansas City, Missouri, United States of America; 5 Department of Obstetrics and Gynecology, Sahlgrenska Academy, University of Gothenburg, Gothenburg, Sweden; Department of Genetics and Bioinformatics, Area of Health Data and Digitalisation, Norwegian Institute of Public Health, Oslo, Norway; 6 Department of Biological Sciences, Vanderbilt University, Nashville, Tennessee, United States of America; 7 Department of Pediatrics, University of Iowa, Iowa City, Iowa, United States of America; 8 Department of Biostatistics, University of Iowa, Iowa City, Iowa, United States of America; 9 Department of Epidemiology, College of Public Health and Department of Pediatrics, Carver College of Medicine, University of Iowa, Iowa City, Iowa, United States of America; 10 Institute of Public Health, University of Southern Denmark, Odense, Denmark; 11 23andMe, Inc. Mountain View, California, United States of America; 12 Obstetrics and Gynecology, University of Helsinki and Helsinki University Hospital, Helsinki, Finland; 13 Rady Children’s Institute for Genomic Medicine, Rady Children's Hospital, San Diego, California, United States of America; Stanford University School of Medicine, UNITED STATES

## Abstract

Preterm birth is a leading cause of morbidity and mortality in infants. Genetic and environmental factors play a role in the susceptibility to preterm birth, but despite many investigations, the genetic basis for preterm birth remain largely unknown. Our objective was to identify rare, possibly damaging, nucleotide variants in mothers from families with recurrent spontaneous preterm births (SPTB). DNA samples from 17 Finnish mothers who delivered at least one infant preterm were subjected to whole exome sequencing. All mothers were of northern Finnish origin and were from seven multiplex families. Additional replication samples of European origin consisted of 93 Danish sister pairs (and two sister triads), all with a history of a preterm delivery. Rare exonic variants (frequency <1%) were analyzed to identify genes and pathways likely to affect SPTB susceptibility. We identified rare, possibly damaging, variants in genes that were common to multiple affected individuals. The glucocorticoid receptor signaling pathway was the most significant (p<1.7e-8) with genes containing these variants in a subgroup of ten Finnish mothers, each having had 2–4 SPTBs. This pathway was replicated among the Danish sister pairs. A gene in this pathway, heat shock protein family A (Hsp70) member 1 like (*HSPA1L*), contains two likely damaging missense alleles that were found in four different Finnish families. One of the variants (rs34620296) had a higher frequency in cases compared to controls (0.0025 *vs*. 0.0010, p = 0.002) in a large preterm birth genome-wide association study (GWAS) consisting of mothers of general European ancestry. Sister pairs in replication samples also shared rare, likely damaging *HSPA1L* variants. Furthermore, *in silico* analysis predicted an additional phosphorylation site generated by rs34620296 that could potentially affect chaperone activity or HSPA1L protein stability. Finally, *in vitro* functional experiment showed a link between HSPA1L activity and decidualization. In conclusion, rare, likely damaging, variants in *HSPA1L* were observed in multiple families with recurrent SPTB.

## Author summary

Preterm birth is the leading cause of infant mortality, and prematurity is further associated with serious morbidities in later life. Genetic and environmental risk factors play a role in the susceptibility to preterm birth. Despite numerous studies, the genetic basis for preterm birth remains poorly defined. We investigated the presence of rare, possibly risk associated nucleotide variants in mothers with spontaneous preterm births (SPTB). The first set of mothers with family history of recurrent preterm births was of northern Finnish origin. An additional set of mothers (sister pairs, both giving birth preterm) of European origin was also studied. Whole exome sequencing identified multiple rare, likely damaging *HSPA1L* variants in several families affected by SPTB, and this gene was associated with the glucocorticoid receptor signaling pathway. Potential involvement of one of the *HSPA1L* variants in SPTB was further supported by large GWAS dataset. In addition, this variant alters protein post-translational modification potential, and thus may affect protein stability and its function as a chaperone.

## Introduction

Preterm birth (PTB), defined as birth before 37 completed weeks of gestation, is a major global public health concern. Worldwide, over 15 million infants (more than one in ten babies) are born preterm and of those, more than one million die from complications related to preterm birth each year [1]. Preterm birth and its complications are the leading cause of neonatal deaths and have become the major cause of death among children under five years old [2]. Moreover, preterm infants are at increased risk, not only of short-term complications but also of life-long disabilities, such as respiratory and cognitive disorders [1]. Preterm birth also increases the risk of adult-onset disorders, such as obesity, diabetes and cardiovascular diseases [3, 4]. Currently, there is no generally effective method for prevention of preterm delivery.

The majority (~70%) of preterm births occur after spontaneous onset of labor, with or without preterm prelabor rupture of the membranes (PPROM) [5]. Most spontaneous preterm births (SPTBs) are idiopathic [1, 5]; however, recurrence of preterm birth among mothers and within families indicates that genetic factors may be important. Genetic factors are estimated to account for 25–40% of the variation in birth timing [6], with the maternal genome playing the major, but not only, role in predisposition to preterm birth [7–11]. Despite many studies of the genetics of SPTB [6, 12, 13], only a few variants have been robustly associated with this outcome [14], and their functional implications are unclear.

Previous genome-wide association studies (GWAS) of SPTB have involved common variants, but they explain only a small portion of the genetic risk. The role of rare variants in SPTB has been essentially unexplored. Whole exome sequencing (WES) in families offers a comprehensive method to identify rare variant associations with disease, including almost complete coverage of the protein coding regions of the genome. Even though studies of rare variants underlying Mendelian disorders have revealed novel genes [15, 16], using WES to study complex multifactorial syndromes remains a challenge [17]. Previous sequencing studies of PTB [18] or PPROM [19, 20] have focused only on a set of candidate gene regions and, consequently, have missed the majority of the coding regions of the genome. In contrast to whole genome sequencing, WES is more cost effective and has the advantage of providing more easily interpreted results.

We performed a WES study using families under the hypothesis that familial recurrence is influenced by rare variants with large individual effects on SPTB susceptibility. Such an approach has the potential of identifying genes containing rare variants shared in these multiplex families, as well as genes in pathways common across families. This method applies a hypothesis-free testing approach to identify potentially novel candidate genes for SPTB.

## Results

### Ingenuity variant analysis and pathway analysis

**Fig 1:**
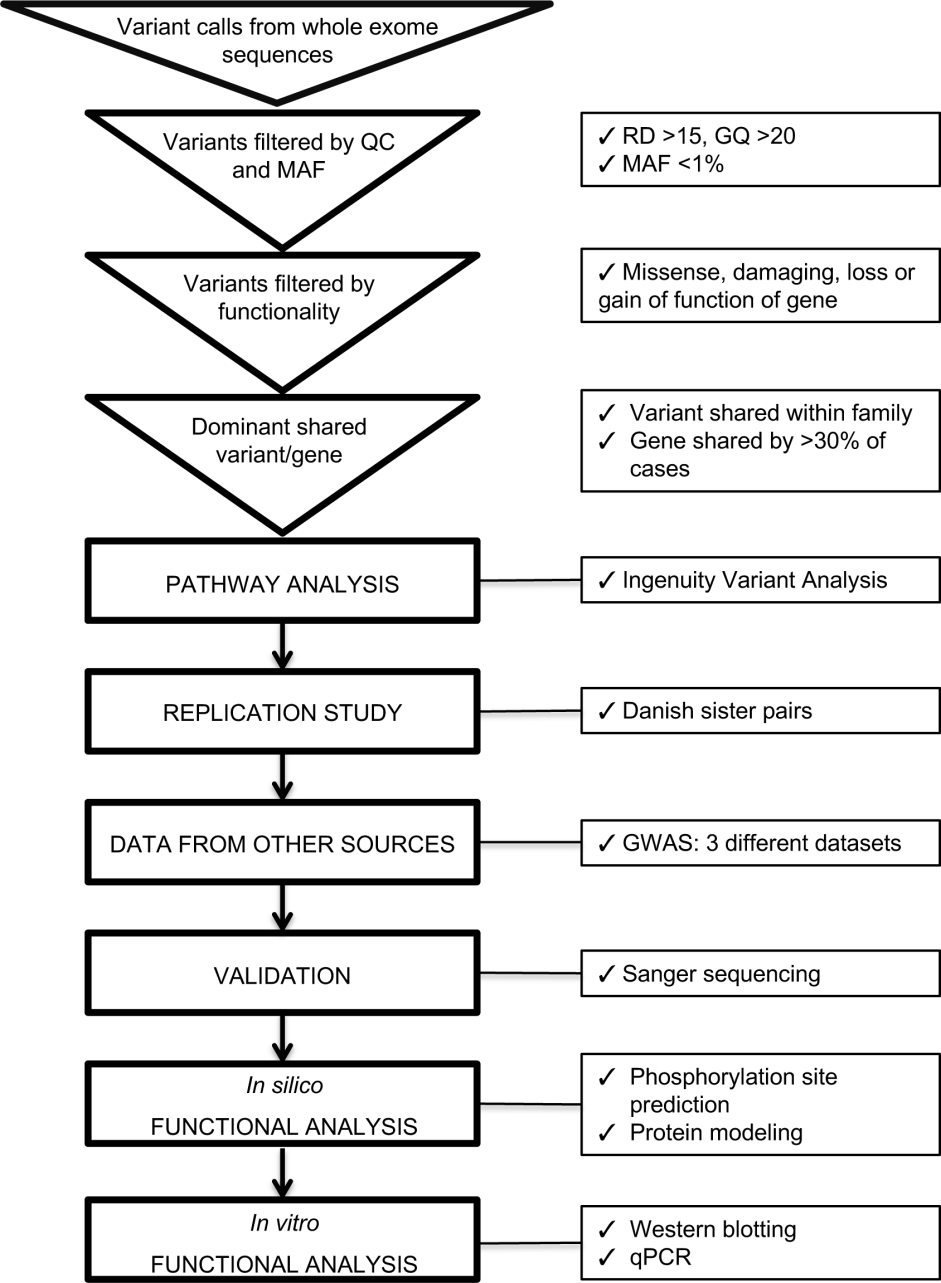
Overview of the whole exome sequencing study workflow. Abbreviations used QC; quality control, MAF; minor allele frequency, RD; read depth, GQ; genotype quality.

Seventeen mothers from seven northern Finnish multiplex families (Discovery cohort) and an additional 192 mothers from 95 Danish families (Replication cohort) were sequenced using WES. The pedigrees of the multiplex Finnish families are shown in S1 Fig. For the Discovery cohort, all samples (except one that was excluded from subsequent analyses) passed the quality control parameters used for the clinical exome sequencing at the CMH; quality control cutoffs were 85% reads aligned, 80% aligned with alignment quality of 20 or greater. For these samples, the mean and median heterozygous/homozygous variant ratios were 0.765 and 0.748, respectively. Prior to variant filtering in Ingenuity, the mean/median numbers of nucleotide variant calls per individual were 318,767/326,474 (Discovery cohort) and 221,682/184,381 (Replication cohort). This difference in variant calls between the Discovery and Replication populations is likely due the fact that populations were sequenced using different Next Generation Sequencing platforms, Illumina for Discovery cohort and Complete Genomics for Replication cohort, and their respective primary quality control measures and variant calling methods were thus different. Mean transition/transversion (Ti/Tv) ratios were 2.2 and 2.0 for Discovery and Replication exomes, respectively. An overview of the WES workflow is presented in Fig 1.

#### Discovery population

An initial nucleotide variant analysis (Ingenuity) was performed in ten mothers (out of 17) with at least two recurrent spontaneous preterm deliveries. These mothers were selected for the first analysis because they represent the most severe cases, i.e., multiple preterm deliveries. We further required that at least three of the cases (30%) had a rare variant, that passed the quality control and prioritizing filters (e.g. likely damaging heterozygous variants), in the same gene. For our purposes, rare variants are defined as minor allele frequency (MAF) <1% in 1000 Genomes, ExAC or in NHLBI ESP. Requiring that at least 30% of the cases had a variant in the same gene, ensured that identified genes are present in cases from at least two different families. This analysis yielded 1,510 different variants in 406 genes. Pathway analysis of these variants in the Ingenuity Variant Analysis identified 64 pathways with p<0.01 under a maternal dominant genetic model (S1 Table). The three most significant pathways (p≤9.80e−7), observed in all ten mothers, were the glucocorticoid receptor signaling, the estrogen receptor signaling and the AMPK signaling pathways. The genes mapping to these pathways are shown in Table 1; many of these genes were present in more than one pathway. Furthermore, when we included the 13 mothers with spontaneous singleton preterm deliveries, and raised the requirement for candidate genes (harboring rare variants) to be shared by at least four mothers, thereby requiring representation in at least two different families, the same three pathways remained within the top five most significant pathways (for glucocorticoid receptor signaling, estrogen receptor signaling and AMPK signaling pathways, the p-values were 3.51e−8, 2.75e−6 and 2.99e−8, respectively).

**Table 1:** Most significant pathway results for Finnish mothers with recurrent SPTB (n = 10).

Pathway name	Pathway p-value^1^	No. of genes	No. of variants	Genes within the pathway identified by WES
Glucocorticoid receptor signaling	1.673E-8	13	36	*AR*, *FOXO3*, *HSPA1L*, *KAT2B*, *MAP3K1*, *NCOA3*, *NCOR1*, *NCOR2*, *NR3C2*, *PIK3R1*, *POLR2J2*/*POLR2J3*, *SMARCA2*, *TBP*
Estrogen receptor signaling	5.067E-7	8	27	*CTBP2*, *KAT2B*, *MED15*, *NCOA3*, *NCOR1*, *NCOR2*, *POLR2J2*/*POLR2J3*, *TBP*
AMPK signaling	9.804E-7	10	28	*AK2*, *CHRNA3*, *FOXO3*, *KAT2B*, *MLST8*, *PFKM*, *PIK3R1*, *PPP2R2B*, *SMARCA2*, *TSC2*

^1^P-value is calculated using a Fisher’s Exact Test based on the distinct sets of genes at the currently-selected filter cascade step, and those that are known to be associated with a given pathway. P-values <0.05 indicate that there are significantly more genes within the pathway than expected by chance.

We used a family-based analysis with a dominant genetic model with at least two affected individuals within a family (five families) for additional Ingenuity Variant Analysis. Within a family, affected mothers shared on average 444 variants (range 278–691) in 243 genes (range 173–381). Individual pathway analysis of each family revealed the glucocorticoid receptor signaling pathway as common to all families and the estrogen receptor signaling pathway in three families with p<0.01 (S2 Table). Several genes in the glucocorticoid receptor signaling pathways (*AR*, *HSPA1L*, *NCOA3* and *NCOR2*) and in the estrogen receptor signaling pathways (*NCOA3* and *NCOR2*) were common to more than one family. These genes all had rare variants likely to be disease causative [21], and were present at a relatively low frequency in the CMH database that was used as internal control data. This analysis only identified two families with p<0.01 for the AMPK signaling pathway, and none of the genes in this pathway were common to more than one family or passed further requirements relative to the control dataset.

#### Replication population

The Ingenuity Variant Analysis of the replication samples (93 sister pairs) had an average of 807 (range 504–1553) rare nucleotide variants in 593 genes (range 357–1112) shared by the two sisters within each family and assumed to be dominantly acting. The glucocorticoid receptor signaling and estrogen receptor signaling pathways were observed in 75 and 79 families with combined median p = 0.0003 and p<0.0001, respectively (S3 Table). Within these two pathways, the genes from the Discovery analyses (*AR*, *HSPA1L*, *NCOA3* and *NCOR2*) were found in between 3 and 12 families from the Replication dataset (Table 2). In an additional two Danish families with affected sister triads, there were 39 common pathways to the two families; the estrogen receptor signaling pathway had the second lowest average p-value (p = 0.0003) and the glucocorticoid receptor signaling pathway had the fifth lowest average p-value (p = 0.0009).

**Table 2:** Pathway results for Danish sister pairs (n = 93); showing only genes related to the Discovery findings.

Rank	Pathway name	No. of Families	Average p-value	Median p-value	Gene (no. of families)
7	Estrogen receptor signaling	79	0.00073	0.00004	*NCOA3* (10), *NCOR2* (12)
10	Glucocorticoid receptor signaling	75	0.00124	0.00034	*AR* (3), *HSPA1L* (5), *NCOA3* (10), *NCOR2* (11)

### Comparison of data obtained from different WES software tools and comparison of rare variants shared within families in Discovery and Replication populations

Three software programs (Ingenuity Variant Analysis, Varseq and the CMH Variant Warehouse) were used to assess common shared (by affected mothers per family) rare variants. Only those variants that passed the prioritizing steps with at least two of the annotating software tools were considered valid and are described below (summarized in S4 Table). The benefits of comparing data obtained from multiple software is that it minimized the possibility of picking up falsely called variants that passed quality control filters only by one software. This approach resulted in a total of 844 variants in the Discovery population. For the Replication population, we combined and compared the shared rare variants passing the annotation and prioritizing steps of Ingenuity Variant Analysis and Varseq; a total of 8431 variants passed the filters of both software tools. The CMH Variant Warehouse was not available for the Replication set. For both populations, variants were categorized as loss of function, moderate, or other, according to their predicted consequences, i.e. pathogenicity (S5 Table). We further compared the list of variants resulting from the family-based analyses (as described above) between the Discovery and Replication populations. Numbers of common genes and variants for both populations are shown in S2 Fig. There were 72 rare variants that were found in both populations in 72 genes (S6 Table).

### *HSPA1L* variants associate with preterm birth in GWAS data of 40,000 mothers

Rare single nucleotide variants from *HSPA1L* [heat shock protein family A (Hsp70) member 1 like], identified by the Discovery Ingenuity pathway analysis, were further investigated using imputed GWAS data that also included variants with MAF <1%. In the Discovery set, variants in *AR*, *NCOA3* and *NCOR2* were either CAG repeat length polymorphisms, in-frame deletions or insertions, respectively, and were, therefore, not investigated in the GWAS datasets. Three independent GWAS datasets were used, one of general European ancestry containing more than 40,000 mothers of live births (23andMe dataset) and two from Northern Europe containing 4,600 and 600 mothers (Nordic and northern Finnish datasets, respectively). In the large 23andMe preterm birth GWAS dataset, the minor allele of rs34620296 in *HSPA1L*, which is in the glucocorticoid receptor signaling pathway, was found to be more common in cases than in controls (case frequency 0.0025 *vs*. control frequency 0.0010, p = 0.002; Table 3). This association was also significant for gestational age as a continuous trait (gestational age as weeks; p = 0.0016, effect -0.8238, standard error 0.2608). The *HSPA1L* variants from the Discovery (rs34620296 and rs150472288) and the Replication (rs482145, rs139193421) analyses are listed in detail in Table 3. In the two smaller GWAS datasets, however, these four *HSPA1L* variants were absent or not significant. Lack of significance may be due to smaller numbers of individuals, especially in cases.

**Table 3:** *HSPA1L* variants in the large GWAS data and their association to SPTB.

Rs#	Chr	Position^1^	Alleles	Protein change	MAF in EA^2^	GWAS^3^ case/control frequencies	GWAS^3^ p-value
rs34620296	6	31778948	C/T	Ala268Thr	0.0014	0.0025/0.0010	0.0022
rs150472288	6	31779089	C/T	Val221Ile	0.0001	Not present	-
rs482145	6	31778314	A/G	Thr479Met	0.0000	0.0000/0.0002	NS
rs139193421	6	31779723	T/C	Ile9Met	0.0002	Not present	-

^1^position in GRCh37.p13.

^2^Frequency for European American (EA) population according to ESP6500: NHLBI GO Exome Sequencing Project (ESP), Exome Variant Server (http://evs.gs.washington.edu/EVS/).

^3^GWAS set including over 40,000 mothers. Association to SPTB; mother investigated as affected i.e. giving birth preterm.

### Sanger sequencing to validate *HSPA1L* variants and to genotype additional family members

Sanger sequencing confirmed the genotypes of the two rare *HSPA1L* missense variants (rs34620296 and rs150472288) in the samples from the Discovery cohort. The rare *HSPA1L* variants were observed in a total of six mothers from four unrelated families. Additional family members with available DNA were sequenced for these variants. Interestingly, in two of the families, female carriers of the maternally inherited rs34620296 minor T-allele were born preterm, whereas in the other two unrelated families the male carriers of maternally inherited rs150472288 minor T-allele were born preterm. However, numbers of minor allele carriers are too small for any definite gender related conclusions.

### Assessing the potential functional impact of the rare *HSPA1L* variants

Pathogenicity predictions for rs34620296 and rs150472288 derived from the Discovery cohort as well as for rs482145 and rs139193421 from the Replication cohort were assessed using *in silico* tools SIFT and PolyPhen-2, and all these variants were predicted as damaging and probably/possibly damaging, respectively (Table 4). In addition, MutationTaster and MutationAssessor predicted all four variants as disease causing and predicted functional (high), respectively. According to the Combined Annotation Dependent Depletion (CADD) score (>20), all of these variants, except for rs139193421, are among the top 1% of deleterious variants in human genome (Table 4).

**Table 4:** Functional predictions for rare missense *HSPA1L* variants.

*HSPA1L* variant	CADD Score^1^	*In silico* prediction of functional effect^2^		RegulomeDB	HaploReg v4.1	
		SIFT	PolyPhen2	Chromatin State: (Example tissue/cell line)	Tissue/cell line: (Active chromatin state^3^^a,b^ or Enriched promoter Histone marks^3^^c,d,e,f^)	DNAse
rs34620296	29.7	Damaging	Probably damaging	Active TSS^5^^a^ (Hela-S3)^4^ Strong transcription (e.g. T cell subtypes^5^, ovary, fetal adrenal gland)	HeLa-S3 (a, d, e, f); Primary T cell subsets^5^ from peripheral blood (c, d, e)	NA
rs150472288	26.4	Damaging	Probably Damaging	Strong transcription (e.g. T cell subtypes^5^, ovary, fetal adrenal gland)	Primary T cell subsets^5^ from peripheral blood (c, d, e)	Ovary
rs482145	28.1	Damaging	Probably damaging	Active TSS (Foreskin Fibroblast Primary Cells) Strong transcription (e.g. T cell subtypes^5^, ovary)	Foreskin Fibroblast Primary Cells (a, b, c, d, e); Primary T cell subsets^5^ from peripheral blood (c, d, e)	NA
rs139193421	NA	Damaging	Possibly damaging	NA	Strong transcription enhancers, Genic enhancers, Enhancers and histone marks (c, d, e) in Primary T cell subsets^5^ from peripheral blood	Psoas Muscle

^1^CADD Score >20 = variant is amongst top 1% of deleterious variants in human genome

^2^*In silico* functional effect predictions: SIFT and PolyPhen-2 annotated by Varseq

^3^a:Active TSS (TssA), active transcription start site, b:Poised Promoter (PromP), c:H3K4me1_Enhancer, d:H3K4me3_Promoter, e:H3K27ac_Enhancer, f:H3K9ac_Promoter

^4^HeLa-S3 = HeLa-S3 Cervical Carcinoma Cell Line

^5^Primary T cell subsets included e.g. effector/memory, regulatory, helper (memory and naïve), CD8+ cells.

To assess potential consequences of these variants on transcriptional activity, we evaluated them for evidence of histone modification or DNase I hypersensitivity. *In silico* tools HaploReg 4.1 and/or RegulomeDB showed that all four variants were in regions that had histone marks, as well as strong transcriptional regulatory signatures in various cells of the immune system, especially in T lymphocytes from peripheral blood (Table 4). Evidence of an active transcription start site was predicted in the HeLa-S3 Cervical Carcinoma Cell Line for rs34620296 and in foreskin fibroblast primary cells for rs482145 (Table 4). Further evidence of active DNA accessibility (DNAse) was found in ovarian tissue for rs150472288, and in psoas muscle tissue for rs139193421 (Table 4). There was also evidence of a transcriptional effect of rs34620296 and rs150472288 (Discovery) in ovary and fetal adrenal gland. Together these results from HaploReg 4.1 and RegulomeDB provide evidence for the potential involvement of *HSPA1L* variants in the endocrine system, as well as in the adaptive immune cells. These variants could, therefore, have a role in the etiology of SPTB.

**Fig 2:**
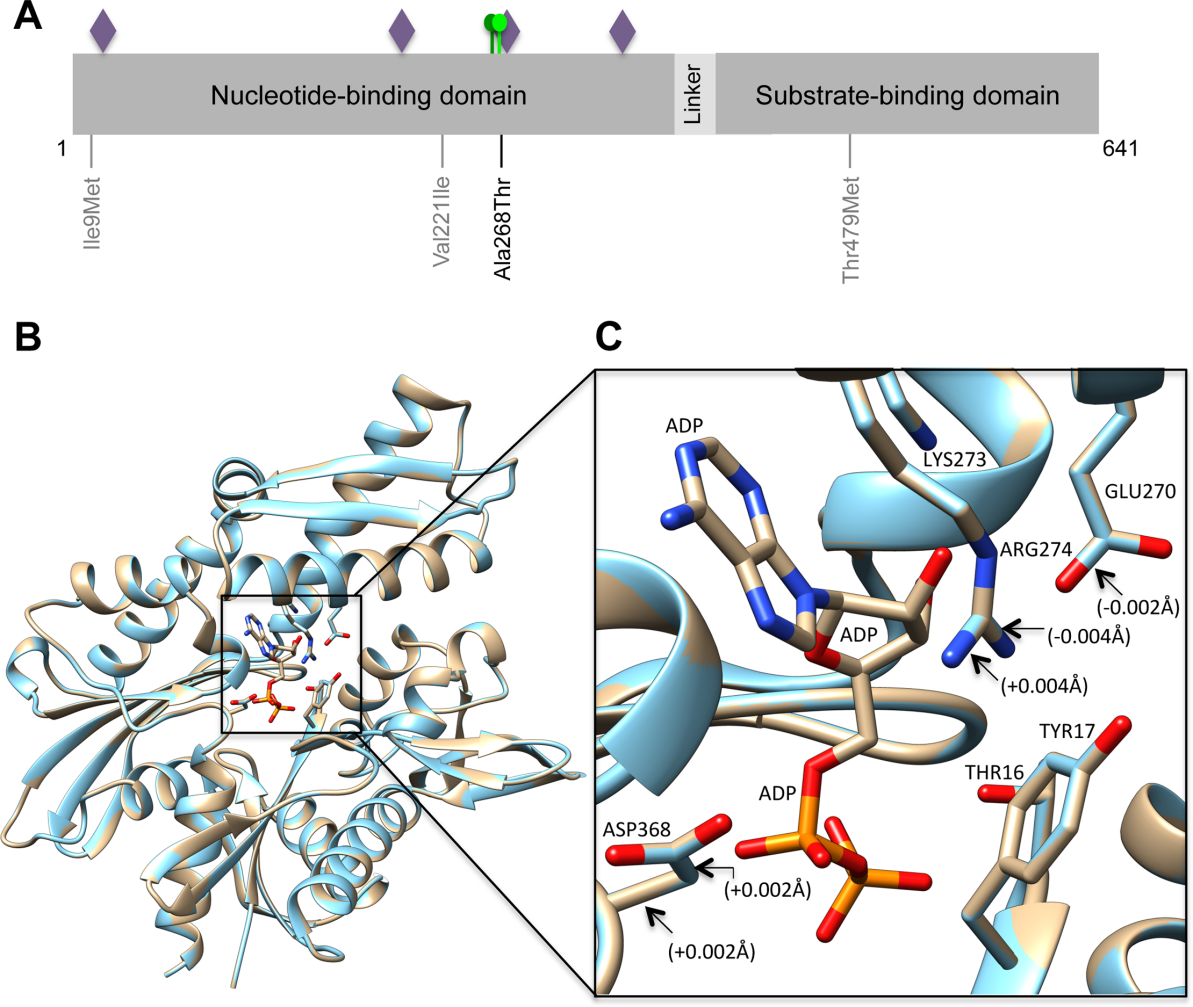
Effects of *HSPA1L* variants on protein sequence and structure. (A) HSPA1L is a 641-amino acid protein that consist of two major functional domains; an N-terminal nucleotide-binding domain and a C-terminal substrate-binding domain, that are connected with interdomain linker. Locations of four *HSPA1L* variants from whole exome sequencing (Discovery and Replication findings) are shown in the protein sequence. Purple diamonds represent ATP nucleotide-binding sites at positions 14−17, 204−206, 270−277 and 341−344. Green circles denote the additional phosphorylation site generated by Ala268Thr and the existing one T267-p. (B) *In silico* comparison of higher order assembly of reference and modified (Ala268Thr) HSPA1L protein models containing an ADP molecule. Overlayed reference and Ala268Thr molecules are presented as gold and light blue rounded ribbon structures, respectively. The interacting ADP molecule is shown as a stick model. (C) Closeup view of the intermolecular contact interface of HSPA1L bound to the ADP molecule. Key interacting residues (amino acids) are shown and their corresponding side chains are presented as stick molecules; nitrogen and oxygen atoms are indicated in blue and red, respectively. All interacting residues; THR16, TYR17, GLU270, LYS273, ARG274, SER277 (not shown in figure) and ASP368, that bind to the ADP ligand showed small changes in the chemical bond lengths. Only changes of ≥0.002Å are shown in the figure (+/- lenght of Ala268Thr structure in relation to reference structure).

We further investigated putative effects of *HSPA1L* rs34620296 on protein structure. This variant was selected due to its association with SPTB in the large 23andMe GWAS dataset. This variant causes an amino acid change from Alanine to Threonine at position 268 (Ala268Thr). According to the NetPhos 3.1 *in silico* prediction, Ala268Thr generates an additional phosphorylation site next to an existing phosphorylation site (T267-p) (S3 Fig). Furthermore, Ala268Thr is near an adenosine triphosphate (ATP) nucleotide-binding site located downstream at position 270−277 (Fig 2A). Gain of phosphorylation may cause changes in binding energy, modulate physio-chemical properties or stability kinetics and dynamics of the protein functions such as strength of protein-protein interactions [22].

To investigate the possible effects of the missense variant on protein structure, the reference HSPA1L protein structure and a structure including the Ala268Thr variant were compared simultaneously using UCSF Chimera. There was not a visible change in the overlaid protein structures (Fig 2B). Instead, there was a slight change in the chemical bond lengths (≥0.002Å) of the adenosine diphosphate (ADP)-ligand binding amino acid side chains at positions Glu270, Arg274 and Asp368, shown in the 3D model of the HSPA1L (Fig 2C). This may be due to the change from a small size, and hydrophobic, (Ala) to medium size, and polar, (Thr) residue. Such a change in the amino acid side chains could affect the binding efficiency of the ADP molecule.

### Tissue expression

To further explore possible underlying biological functionality, we investigated the tissue expression established via *HSPA1L*, along with *AR*, *NCOA3* and *NCOR2*, using HumanBase (http://hb.flatironinstitute.org). *HSPA1L* was expressed in placental tissue with reasonable confidence (0.65), and in ovarian (0.57) and fetal tissues (0.48) as well as in uterus (0.29). For *HSPA1L*, *AR*, *NCOA3* and *NCOR2* together, the average expression confidence was high in placenta (0.74), ovary (0.70), fetus (0.65), and moderate in uterus (0.29), indicating high confidence for expression in female reproductive system overall (S4 Fig).

### Functional consequences of the *HSPA1L* Ala268Thr variant

**Fig 3:**
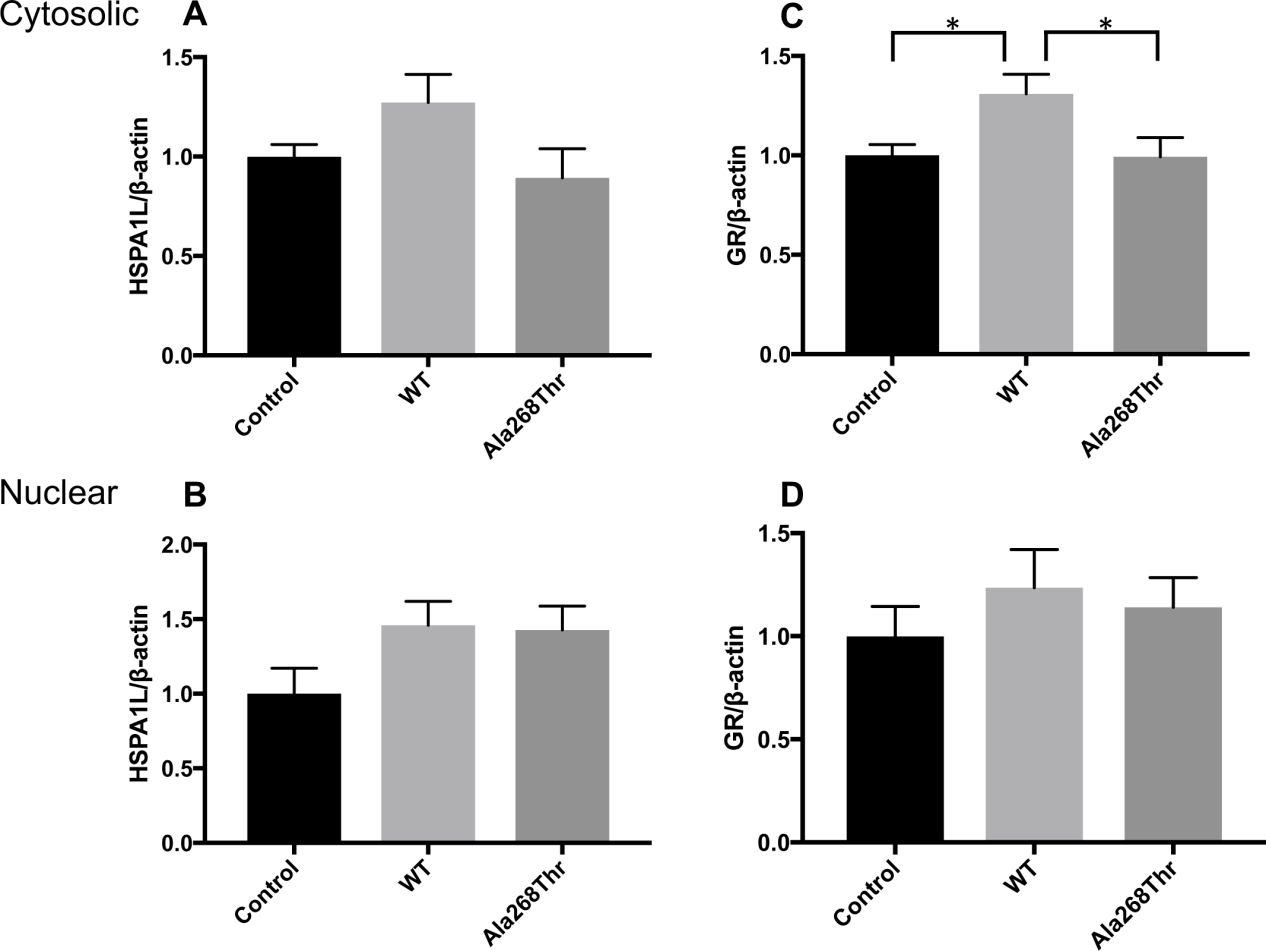
HSPA1L and GR protein levels in decidualized human endometrial stromal fibroblasts. Cultured ESFs were transfected with WT or Ala268Thr *HSPA1L*-pcDNA3.1 constructs or with empty pcDNA3.1 vector (control). Cells were treated with decidualization media supplemented with 100nM dexamethasone (glucocorticoids) for 72h. Both cytosolic and nuclear protein were extracted, and HSPA1L and GR protein levels were measured by Western blot. Band intensity of HSPA1L or GR was normalized to band intensity of the corresponding β-actin. Cytosolic (A) and nuclear (B) HSPA1L levels as well as cytosolic (C) and nuclear (D) GR levels are shown for control (empty vector), WT and Ala268Thr sample groups. Each experiment was performed as triplicates in three different passages (n = 9 each group, except n = 8 for nuclear control group) and bars represent mean + SEM. Significant p-value <0.05 is presented with an asterisk. Cytosolic GR levels were significantly higher (p = 0.04) in the WT compared to the Ala268Thr group as well as in the WT compared to the control group (p = 0.04).

To determine whether the *HSPA1L* Ala268Thr (rs34620296) variant alters activity of the GR signaling pathway, we analyzed the consequences of glucocorticoid exposure during decidualization. Human endometrial stromal fibroblasts were transfected with plasmids containing either WT or Ala268Thr cDNA, or with empty vector serving as control. The cells were treated with decidualization media for 72h in a presence of glucocorticoids (100nM dexamethasone) as a surrogate of stress. Protein levels of HSPA1L and GR, as well as mRNA levels of Wnt Family Member 4 (*WNT4*) were measured. Cells transfected with the WT *HSPA1L*-pcDNA3.1 trended to greater increases in cytosolic HSPA1L protein content than those transfected with the Ala268Thr *HSPA1L*-pcDNA3.1 (mean ± SEM; 1.272 ± 0.142 *vs*. 0.893 ± 0.146, respectively, p = 0.09) (Fig 3). Furthermore, the Western blot analysis showed that the relative cytosolic protein levels of GR differed significantly between the WT and Ala268Thr groups with more GR present in the WT group than in the Ala268Thr group (mean ± SEM; 1.309 ± 0.099 *vs*. 0.993 ± 0.096, respectively, p = 0.04) (Fig 3; numerical data available in S7 Table).

Next, we determined the relative gene expression of *WNT4* by qPCR. *WNT4* is a critical decidualization target found in the recent GWA study [14] associated with gestational length. Increased expression of *WNT4* was observed in the WT group, whereas, the Ala268Thr group was less able to activate the WNT4-signaling pathway leading to a lower expression of *WNT4* (p = 0.04).

## Discussion

To move beyond traditional case-control GWAS and family-based linkage studies, we performed a case-only whole exome sequencing study designed to investigate the burden of rare variants in families with recurrent SPTB. Whole exome sequencing enables the discovery of rare, putatively functional variants associated with the etiology of complex disease on a gene-by-gene or a pathway-by-pathway basis, and enrichment in multiplex families provides a means to filter large-scale sequencing data.

Comparisons of mothers with recurrent preterm deliveries identified the glucocorticoid receptor signaling pathway as a candidate for mediating the risk of SPTB. Specifically, within this pathway, likely pathogenic missense variations in *HSPA1L* were found among four unrelated Finnish families (rs34620296 and rs150472288), and within Danish sister pairs (rs482145 and rs139193421). Notably, the rs34620296 minor allele variant was observed at a higher frequency in cases than controls in a very large 23andMe GWAS set. These variants were also identified via bioinformatics analyses as likely affecting either protein function or expression. Further functional evidence linked HSPA1L activity and decidualization.

*HSPA1L* is a member of the Hsp70 superfamily and is near *HSPA1A* and *HSPA1B* within the major histocompatibility complex class III region on chromosome 6. The HSPA1L protein (also known as Hsp70-hom) is ~90% identical to HSPA1A and HSPA1B, also known as Hsp70-1 and Hsp70-2, respectively [23, 24]. Heat shock proteins (HSPs) are highly conserved cellular defense mechanisms for cell survival and are present in all cell types in all organisms. Some HSPs are expressed constitutively, while others are stress-induced (e.g. heat, hypoxia, oxidative stress, infection and inflammation) [25, 26]. Intracellular HSPs act as molecular chaperones and, together with co-chaperones, stabilize existing proteins against aggregation, mediate folding of newly translated proteins, and assist in protein translocation across intracellular membranes [25, 27]. HSPs are categorized into families according to their approximate molecular weight; of which Hsp70 (a group of proteins sized approximately 70 kDa) is the best characterized. Potential involvement of stress-induced HSPA1A in adverse pregnancy outcomes, including preeclampsia and PTB, has previously been suggested [28, 29]. Although, studies of the role of HSPA1L and *HSPA1L* in pregnancy are lacking, there is some evidence of involvement in adverse pregnancy outcomes such as preeclampsia [30].

The rare *HSPA1L* missense variants observed in our study, are in the nucleotide-binding domain (NBD), except the rs482145, which is in the substrate-binding domain (SBD) (Fig 2). ATP binds to the NBD, which is followed by the exchange from low-binding affinity ATP state to high-binding affinity ADP state [29, 31, 32]. We showed that the non-synonymous variant rs34620296 (Ala268Thr) generates an additional phosphorylation site near the nucleotide-binding site. It showed a modest change in the binding efficiency at this site, which could affect the interaction with ADP or HSPA1L stability itself, as suggested by our transfection studies. In agreement with our findings, a previous study of Caucasian patients with inflammatory bowel disease found that rare mutations in *HSPA1L* were significantly enriched in patients but absent in healthy controls [33]. Interestingly, one of the associated rare variants was Ala268Thr, and further *in vitro* biochemical assays of the recombinant HSPA1L showed reduced chaperone activity with this variant [33]. There is also evidence that possibly connects inflammatory bowel disease to adverse perinatal outcomes [34]. Additionally, a previous SPTB study in African Americans found a common nonsynonymous *HSPA1L* variant, rs2075800, to associate with SPTB [35]. Furthermore, a meta-analysis of previously PTB associated genes linked *HSPA1L* and SPTB using Ingenuity Pathway Analysis [36].

Due to a very low incidence of the rare *HSPA1L* variants associating with SPTB in our study, the anticipated attributable risk in the population level is probably small. However, the identification of the damaging alleles may facilitate the identification of causative pathways. For instance, interaction between Hsp70 and Hsp90 chaperones as well as their co-chaperones is essential in the maturation and inactivation of nuclear hormone receptors (e.g. glucocorticoid, androgen, estrogen and progesterone receptors) [37, 38]. In the absence of its ligand, glucocorticoid receptor (GR) is bound to a complex constituting of Hsp40, Hsp70 and Hsp90 chaperones; this complex keeps the GR in a ligand-receptive conformation but remaining transcriptionally inactive until ligand binding [38]. As shown previously [33], rare *HSPA1L* variants can cause partial loss of HSPA1L chaperone activity, and therefore, altered function or expression. Altered function of the chaperones can compromise the stability of the GR complex, leading to an accumulation of partially unfolded proteins that are prone for aggregation and degradation events [37].

Glucocorticoids, steroid hormones that mainly signal through the GR, have anti-inflammatory and immunosuppressive actions. Glucocorticoid signaling communicates with estrogen signaling pathways to tightly regulate the pro- and anti-inflammatory milieu in reproductive tissues [39], and progesterone signaling, *via* nuclear GR, mediates anti-inflammatory and immunosuppressive effects in genital tract during pregnancy [40, 41]. Sustaining a pregnancy is a complex interplay and balance between the innate and adaptive immune cells in the reproductive tissues and at the maternal-fetal interface. Imbalance between the inflammatory cells can cause a breakdown of maternal-fetal tolerance leading to activation of labor (both term and preterm). An untimely stimulus (e.g. stress, infection or inflammation) together with impairments in the glucocorticoid receptor signaling pathway could impose an inadequate response against inflammation or stress. This can elicit a shift from an anti-inflammatory to pro-inflammatory microenvironment, causing a premature activation of labor initiating signals resulting in preterm birth [42, 43].

Possible limitations of our study are that the Discovery and Replication populations were sequenced using different Next Generation Sequencing platforms, and primary quality control measures and variant calling methods were thus different. In addition, Next Generation Sequencing generates an enormous amount of data, which could lead to many sequencing artifacts that may be misidentified as variants. We attempted to minimize these artifacts by applying a variety of quality control filters and using a large internal control population to detect potential sequencing or annotation errors. We also compared the results of variant annotation and prioritizing filters from three different software tools to ensure reproducible results. Furthermore, reported variants were confirmed by Sanger sequencing. Another possible limitation was that our study did not include unrelated control samples. This limitation has been partly overcome with the use of additional large GWAS datasets including control samples.

In conclusion, whole exome sequencing of families with recurrent occurrence of SPTB enables identification of rare alleles influencing the predisposition to SPTB. Among the individual genes, two minor alleles of *HSPA1L* had a strong association to SPTB in multiplex Finnish families and the association of a specific minor allele was confirmed in a large GWAS set. Furthermore, this variant was associated with altered modification and function of the protein. Overall, our data suggest the need for precise regulation of steroid signaling in mediating birth timing.

## Materials and methods

### Ethics statement

Written informed consent was obtained from all individuals participating in this study, and the study was approved by the Ethics committees of the participating centers: Oulu University Hospital (78/2003, 73/2013), University of Southern Denmark (NVK#1302824), and University of Iowa (IRB#200608748). Individuals in the large European American GWAS were research participants of 23andMe, Inc., a personal genetics company. All 23andMe participants provided informed consent and participated in the research online, under a protocol approved by the external AAHRPP-accredited IRB, Ethical & Independent Review Services (E&I Review).

### Study populations and inclusion criteria for SPTB cases

#### Discovery population: Northern Finnish multiplex families

A total of 17 mothers from seven northern Finnish families were subjected to whole exome sequencing. This included 13 mothers with spontaneous preterm deliveries, of whom 10 had recurrent (2–4) spontaneous preterm deliveries at less than 36 weeks of gestation. The remaining mothers were grandmothers (mothers of the affected index mothers) with pregnancy history including extremely preterm twin pregnancy or late preterm pregnancy. Pedigrees of the large families highlighting the mothers selected for this study are presented in S1 Fig. The majority (five out of seven) of these families have been included in our previous linkage studies [44, 45].

Unrelated families with history of recurrent preterm births were selected retrospectively from the birth diaries of Oulu University Hospital from 1973–2003, and prospectively from 2003–2005. Descriptions of inclusion and exclusion criteria have previously been reported in detail [44, 45]. A genealogical study was performed according to published criteria [46], and to reveal close consanguinity or common residence, families were followed up to 17^th^ century using the provincial and national archives of Finland. All families were of the northern Finnish origin. Furthermore, families with apparent maternal inheritance of SPTB were selected for this study. Spontaneous preterm birth was defined as birth before 36 completed weeks of gestation (excluding the borderline 36th week), with delivery occurring with intact membranes or following PPROM. Pregnancies with known risk factors for SPTB were excluded from the primary analyses.

#### Replication population: Danish sister pairs

This population set, recruited from Denmark, included 192 women from 95 families. This cohort consisted of 93 affected sister pairs (both sisters have given birth preterm) and two sister triads with preterm deliveries. Preterm delivery was set to occur before 37 completed weeks of gestation, and all women had experienced at least one PTB. In the majority (83%) of the sister pairs, both sisters had experienced a spontaneous PTB. All individuals were of the European ancestry.

### Whole exome data generation

DNA samples of the 17 individuals from Discovery population were extracted from whole blood and saliva samples using standard methods [45]. Although, using DNA from both blood and saliva samples, there were no major difference in the overall sequencing metrics (alignment metrics or total number of variants) between the sample types. DNA samples were subjected to exon specific next generation sequencing performed at the Center for Pediatric Genomic Medicine, Children’s Mercy Hospital (CMH; Kansas City, MO). Exome samples were prepared with the Illumina Nextera Rapid Capture Exome kit according to the manufacturer’s protocols as described previously [47]. Sequencing was performed on Illumina HiSeq 2500 instruments utilizing v4 chemistry with 2 x 125 nucleotide sequences. Sequence data were generated with Illumina RTA 1.18.64.0 and bcl2fastq-1.8.4, and aligned against the reference human genome (GRCh37.p5) using bwa-mem [48], and variant calls were made using the Genome Analysis Toolkit (GATK) [49] version 3.2–2 using previously described methods [50]. Duplicate reads were identified and flagged with the Picard MarkDuplicates tool. Realignment of reads around known indels was performed with the RealignerTargetCreator and IndelRealigner, and variants were called on individual samples using the HaplotypeCaller modules of the GATK.

In addition, whole exome sequencing was performed on 192 affected individuals from 95 Danish families (Replication set). Exome capture of the samples were carried out with the BGI Exon Kit following manufacturer’s protocols (BGI, Shenzhen, China). DNA libraries were generated using combinatorial Probe Anchor Ligation (cPAL) technology, and 35 base paired end reads were generated from 500 bp genomic fragments. Whole exome sequencing was performed using the Complete Genomics platform (BGI) and using the manufacturer’s pipeline. Reads were aligned against the National Center for Biotechnology Information (NCBI) build 37 reference human genome.

### Variant annotation, filtering and prioritizing

The variant call files (VCF), containing the variant call results, generated by CMH and BGI were analyzed using Ingenuity Variant Analysis Software (Qiagen, Germany) and Golden Helix VarSeq Software v.1.2.1 (Bozeman, MT) for both the Discovery and Replication population sets. Variants were filtered based on variant quality control measurements, frequency and predicted pathogenicity, as well as a dominant inheritance model.

In the Ingenuity Variant Analysis, low quality variants (read depth <15 and call quality <20) were removed. Furthermore, we only included rare variants (i.e. MAF <1% in the 1000 Genomes Project, ExAC or in European American population in NHLBI ESP exomes) and variants that would likely have functional effect (i.e. variants that are predicted by SIFT or PolyPhen-2 as damaging or likely damaging, listed in Human Gene Mutation Database, or associated with gain or loss of function of a gene).

In VarSeq, variants with read depth <15 and genotype quality score <20 were excluded. Only rare variants in Europeans (MAF <1% or absent; 1kG Phase 3: Variant frequencies 5, GHI Jan 2015) and missense or loss-of-function variants were included for analyses. Further filtering was applied to the data obtained from VarSeq. Variant quality was enhanced by applying range criteria (0.3–0.85) for alternative allele frequency (i.e. ratio of alternate allele read depth / alternate allele read depth + reference allele read depth); variants outside this range were excluded. Allele frequencies were searched from the Sequencing Initiative Suomi (SISu) database (www.sisuproject.fi) for variants originating from the Finnish mother data, and from the Exome Aggregation Consortium (ExAC) database (http://exac.broadinstitute.org/) for Danish sister pair data. For these variants, a MAF cut-of value <1% in Finnish general population (SISu) or European “non-Finnish” (ExAC) population was used for Finnish or Danish mothers, respectively. The SISu database was used for Finnish mothers to exclude rare variants that are enriched in Finnish general population compared to the rest of the Europeans.

For the Discovery samples, we also used allele frequency calculations derived from Center for Pediatric Genomic Medicine’s CMH Variant Warehouse database (http://warehouse.cmh.edu) including ~3900 individuals previously sequenced at the center [50]. Pathogenicity was categorized according to the American College of Medical Genetics [21] as 1; previously reported to be disease-causing, 2; expected to be pathogenic (loss of initiation, premature stop codon, disruption of stop codon, whole-gene deletion, frame shifting indel, and disruption of splicing), and 3; unknown significance but potentially disease-causing (nonsynonymous substitution, in-frame indel, disruption of polypyrimidine tract, overlap with 5' exonic, 5' flank, or 3' exonic splice contexts). Only variants that fit one of these criteria (1−3) were included for analyses. Rare and novel variants with relatively high frequency in this internal control population were also excluded as they were thought to be technical artifacts.

### Model of inheritance, gene and variant sharing filters

In Ingenuity Variant Analysis, a dominant inheritance model (including gain of function variants, and all heterozygous, compound heterozygous, haploinsufficient, hemizygous, and het-ambiguous variants) was used to investigate predisposing variants that are inherited in the families. When analyzing affected mothers as a group, rare variants in *genes* that were common for a proportion of all cases were investigated. Whereas in family specific analyses, only *variants* that were shared by the affected individuals within each family were included.

### Pathway analysis

Ingenuity Variant Analysis provides a list of most significant pathways calculated specifically for each filtering output. P-values were calculated according to Fisher’s exact test assessing overlap enrichment of dataset-variant genes relative to known phenotype-implicated genes. Here, only pathways with p<0.01 were included for further analyses.

### Investigation of rare variants in genome-wide association study data

To investigate rare variants in genes arising from the whole exome data in a larger population setting including controls, we used available sources of preterm birth GWAS data. GWAS data from a large cohort, identified among 23andMe’s research participants, included 43,568 mothers of general European ancestry [14] and meta-analysis data including 4,632 mothers from three independent Nordic (Finnish, Danish, and Norwegian) birth cohorts of European ancestry [51]. In addition, a set of GWAS data from a total of 608 mothers passing quality control measures was available. This set included mothers with spontaneous preterm deliveries and mothers with term deliveries originating exclusively from northern Finland. Genotyping was performed with Illumina Human CoreExome chip, followed by prephasing and imputation procedures with ShapeIT2 [52] and IMPUTE2 [53]. Association analysis was performed using SNPTEST v. 2.5.2 [54].

### Resequencing the candidate variants

Since WES methodologies are associated with significant false positive rates, the presence of interesting variant findings from WES analyses was confirmed using Sanger sequencing. Samples were sequenced using capillary electrophoresis with ABI3500xL Genetic Analyzer (Applied Biosystems, CA) in Biocenter Oulu Sequencing Center, University of Oulu, Oulu, Finland. Details of the PCR primers and reaction conditions are available upon request.

### Assessing the functional impact of the rare *HSPA1L* variants using *in silico* modeling

The possible functional effect of the rare *HSPA1L* variants (rs34620296 and rs150472288 from Discovery analyses as well as rs482145 and rs139193421 from Replication analyses) were investigated using *in silico* prediction tools such as SIFT, PolyPhen-2, MutationTaster and MutationAssessor. These pathogenicity predictions were annotated using Varseq, whereas CADD scores to identify pathogenic and deleterious variants were obtained from Ingenuity. Variants with CADD score >20 are amongst top 1% of deleterious variants in human genome [55]. RegulomeDB (http://www.regulomedb.org/) was used to investigate variant locations for e.g. chromatin state activity. In addition, we used HaploReg v4.1 (http://archive.broadinstitute.org/mammals/haploreg/haploreg.php) to assess whether variants are located within regions that show evidence for promoter or enhancer activity (i.e. presence of histone modification marks H3K4me1 and H3K27ac that are associated with enhancer regions, or H3K4me3 and H3K9ca that are associated with promoter regions), as well as for DNase I hypersensitivity in human tissues and cell line samples.

We further investigated the potential effects that missense variation rs34620296 (Ala268Thr) could have on protein sequence or structure. We used NetPhos 3.1 [56] to investigate possible changes in phosphorylation events in HSPA1L sequence. NetPhos 3.1 predicts serine, threonine or tyrosine phosphorylation sites in amino acid sequences of eukaryotic proteins. Evidence of being a phosphorylation site is given when the score is above the threshold (0.5). To investigate the possible effects of missense variant in protein structure, the reference protein structure and the modified protein structure, including the missense variant, were compared. Original (UniProtKB: P34931) and modified (Ala268Thr) amino acid sequences were submitted to SWISS-MODEL (https://swissmodel.expasy.org/) for protein modeling. Resulting protein models were compared simultaneously using UCSF Chimera. Molecular graphics and analyses were performed with the Chimera-1.11.2. package (https://www.cgl.ucsf.edu/chimera/). Chimera was developed by the Resource for Biocomputing, Visualization, and Informatics at the University of California, San Francisco (supported by NIGMS P41-GM103311) [57].

### *In vitro* functional analysis using human endometrial stromal fibroblasts

#### Construction of pcDNA 3.1(+) plasmid with *HSPA1L* inserts

pGEX-6P-1 vector including *HSPA1L* WT or Ala268Thr inserts were generously provided by Dr. Michael Snyder and Dr. Shinichi Takahashi from Stanford University. These vectors were generated as described in their previous study [33]. To obtain a more suitable vector for human derived cells, the *HSPA1L* inserts were cut from the pGEX-6P-1 vector using BamHI-HF (New England Biolabs) and NotI (Invitrogen) restriction enzymes. The *HSPA1L* sequences were subcloned into pcDNA3.1(+) vector (Invitrogen) by ligating into the BamHI/NotI sites using Quick ligase (New England Biolabs). The recombinant plasmids (WT or Ala268Thr) were sequenced to confirm the successful construction before continuing into further functional studies.

#### Cell culture and transfection

Two primary human endometrial stromal fibroblast (ESF) lines (HsESC_217S and HsESC_218S) were obtained from the Hugh Taylor/Clare Flannery labs in the Department of Obstetrics, Gynecology, and Reproductive Sciences at Yale University. HsESC_217S and HsESC_218S were derived from two separate patients each undergoing a polypectomy surgical procedure on days 3 and 9 of the menstrual cycle, respectively. ESFs were cultured in DMEM (GIBCO), supplemented with 10% fetal bovine serum (FBS), 200mM L-glutamine and 1% penicillin-streptomycin (GIBCO) in a humidified incubator at 37°C with 5% CO_2_.

ESF cells were cultured in 6-well plates until confluent and transfected by Lipofectamine 3000 (Thermo Fisher Scientific) with 500ng WT *HSPA1L*-pcDNA3.1(+) or Ala268Thr *HSPA1L*-pcDNA3.1(+) per well and maintained for 24h in OPTI-MEM reduced serum medium (GIBCO). Cells transfected with empty pcDNA3.1(+) vector were used as control. After 24h, the medium was replaced with fresh media (DMEM, 10% FBS, 200mM L-glutamine, 1% penicillin) and cells were let to recover for 24h. To induce decidualization, cells were treated with 0.5mM 8-Br-cAMP (Sigma) and 0.5mM of the progesterone analog, medroxy-progesterone acetate (Sigma), for 72h in DMEM supplemented with 2% FBS, 1% penicillin and sodium bicarbonate. Additionally, this media contained 100nM Dexamethasone (Sigma) to mimic increased stress levels. After the 72h period, cells were harvested using Trypsin (0.05% Trypsin, 0.5mM EDTA, PBS) for either protein or RNA extraction procedures. All treatments were done as 3 replicates in three different passages (n = 9 each group).

#### Western blotting

From the harvested cell pellet, protein was extracted using NE-PER Nuclear and Cytoplasmic Extraction Kit (Thermo Fisher Scientific) according to the manufacturer’s instructions and stored at -80°C until used. Protein content was assessed using Pierce BSA Protein Assay (Thermo Fisher Scientific) according to the manufacturer’s instructions. Denaturated protein samples (20 μg for cytoplasmic or 10 μg for nuclear proteins) were loaded onto NuPAGE 4–12% Bis-Tris gels (Invitrogen) and separated by electrophoresis in 1x NuPAGE MOPS SDS running buffer (Thermo Fisher Scientific). Protein bands were electroblotted onto a nitrocellulose membrane (GE Healthcare) using NUPAGE transfer buffer (Thermo Fisher Scientific) and 20% methanol. After transferring the bands, membranes were blocked in 5% nonfat dry milk in Tris buffered saline (TBS; Fisher Scientific) containing 0.1% Tween 20 (Sigma) for 1h in RT. Membranes were incubated overnight in +4°C with primary monoclonal rabbit antibodies against HSPA1L (dilution 1:1000; GTX111045; GeneTex), GR (dilution 1:1000; #3660S; Cell Signaling Technologies) or, as control, β-actin (dilution 1: 20,000; A5060; Sigma) in 5% nonfat dry milk TBS-0.1%Tween. In the following day, membranes were washed with TBS-0.1%Tween and incubated with horseradish peroxidase conjugated goat anti-rabbit IgG secondary antibody (dilution 1: 10,000; sc-2004; Santa Cruz Biotechnology) for one hour in RT. Membranes were washed with TBS-0.1%Tween, and for luminol based band detection, treated with Super Signal West Pico or Dura Luminol (Thermo Fisher Scientific). Protein band intensities of HSPA1L and GR were quantified relative to corresponding β-actin band (i.e. protein of interest: β-actin ratio) using Image J. The data was tested for normality, and one-way ANOVA in GraphPad Prism v.7.04 (GraphPad Software, Inc.) was used to compare the protein levels. Statistical significance was defined as a p<0.05.

#### RNA extraction, cDNA synthesis and real-time quantitative PCR

Total RNA was extracted using the RNeasy Mini Kit (Qiagen). cDNA synthesis from total RNA was performed using Quantitect Reverse Transcription Kit (Qiagen) according to manufacturer’s instructions. Expression levels of mRNA were determined by real-time quantitative PCR (qPCR) on Step One Plus real-time PCR system (Applied Biosystems). Amplification reactions were performed as duplicate in 20μl final volume containing 25ng cDNA, *WNT4* and *GAPDH* TaqMan gene expression assays (Hs01573505_m1 and Hs02758991_g1, respectively, Thermo Fisher Scientific), and 2x TaqMan Gene Expression Master Mix (Thermo Fisher Scientific). *GAPDH* was used as a housekeeping gene. Relative quantification of *WNT4* was performed using the ΔΔCt method. One-tailed Student’s *t* test was used to compare gene expression levels.

## Supporting information

S1 Fig Pedigrees of the seven northern Finnish multiplex families with recurrent spontaneous preterm births. Circles represent females and squares males; symbols with a line intersecting indicate that individual is deceased. Diamonds denote an unspecified number of infants born at term, i.e. gestational age (GA) ≥37 weeks. Letters above symbols indicate individuals for whom WES data was gathered and an asterisk next to the letter denotes the individual as a recurrent mother. Letters inside brackets indicate grandmothers with term, twin or non-spontaneous deliveries, and were not included in the primary analyses. Pedigrees were created using Progeny Pedigree tool (https://pedigree.progenygenetics.com/).
(PDF)

S2 Fig Comparison of Finnish (Discovery) and Danish (Replication) family analyses. For both the Discovery (total of 5 families) and Replication (total of 93 families) populations, these analyses only included rare variants that passed the filters of multiple annotating software tools and were shared by affected family members. The total number of genes (A) and variants (B) that were common between the two populations are presented in the middle of the Venn diagram.
(TIF)

S3 Fig Phosphorylation site prediction results. The potential effect of Ala268Thr on HSPA1L protein sequence was predicted using NetPhos 3.1. This missense variant generates an additional phosphorylation site on the HSPA1L sequence with high confidence (0.855; above the threshold of 0.5). The T268-p phosphorylation site is marked with an asterisk.
(TIF)

S4 Fig Tissue expression of *HSPA1L*, *AR*, *NCOA3* and *NCOR2* in pregnancy related tissues. (A) *HSPA1L* is expressed with reasonable confidence in placental tissue (0.65), ovarian (0.57) and fetal tissues (0.48) as well as in uterus (0.29). (B) For *HSPA1L*, *AR*, *NCOA3* and *NCOR2* together, the average expression confidence is high in placenta (0.74), ovary (0.70), fetus (0.65), and moderate in uterus (0.29), indicating high confidence for expression in female reproductive system overall. Tissue expression was established using HumanBase (http://hb.flatironinstitute.org).
(TIF)

S1 Table Pathway results for Finnish mothers with recurrent preterm births (n = 10). (DOCX)

S2 Table Pathway results for Finnish families (n = 5) with multiple affected individuals: Only pathways seen in at least two different families are listed. (DOCX)

S3 Table Pathway results for Danish sister pairs (n = 93): only pathways common for ≥20 families are listed. (DOCX)

S4 Table Comparison of variant filtering results from different analysis software used for Finnish families with multiple affected individuals (n = 5). (DOCX)

S5 Table Functional categories of rare variants passing the annotation and prioritizing steps of multiple software tools. (DOCX)

S6 Table Functional categories of rare variants common for both Discovery and Replication populations. (DOCX)

S7 Table Relative HSPA1L and GR protein levels in cytosolic and nuclear extracts; supplementing the Fig 3. (DOCX)

## References

[1] Blencowe H, Cousens S, Chou D, Oestergaard M, Say L, Moller AB, et al Born too soon: the global epidemiology of 15 million preterm births. Reproductive health. 2013;10 Suppl 1:S2. 24625129 10.1186/1742-4755-10-S1-S2 PMC3828585

[2] Liu L, Oza S, Hogan D, Perin J, Rudan I, Lawn JE, et al Global, regional, and national causes of child mortality in 2000–13, with projections to inform post-2015 priorities: an updated systematic analysis. Lancet (London, England). 2015;385(9966):430–40. 10.1016/S0140-6736(14)61698-6 25280870

[3] Sipola-Leppanen M, Vaarasmaki M, Tikanmaki M, Matinolli HM, Miettola S, Hovi P, et al Cardiometabolic risk factors in young adults who were born preterm. American journal of epidemiology. 2015;181(11):861–73. 10.1093/aje/kwu443 25947956 PMC4445394

[4] Tikanmaki M, Tammelin T, Sipola-Leppanen M, Kaseva N, Matinolli HM, Miettola S, et al Physical Fitness in Young Adults Born Preterm. Pediatrics. 2016;137(1). 10.1542/peds.2015-1289 26715606

[5] Goldenberg RL, Culhane JF, Iams JD, Romero R. Epidemiology and causes of preterm birth. Lancet (London, England). 2008;371(9606):75–84. 10.1016/S0140-6736(08)60074-4 PMC7134569 18177778

[6] Bezold KY, Karjalainen MK, Hallman M, Teramo K, Muglia LJ. The genomics of preterm birth: from animal models to human studies. Genome medicine. 2013;5(4):34 10.1186/gm438 23673148 PMC3707062

[7] Clausson B, Lichtenstein P, Cnattingius S. Genetic influence on birthweight and gestational length determined by studies in offspring of twins. BJOG: an international journal of obstetrics and gynaecology. 2000;107(3):375–81. 10740335 10.1111/j.1471-0528.2000.tb13234.x

[8] Kistka ZA, DeFranco EA, Ligthart L, Willemsen G, Plunkett J, Muglia LJ, et al Heritability of parturition timing: an extended twin design analysis. American journal of obstetrics and gynecology. 2008;199(1):43.e1–5. 18295169 10.1016/j.ajog.2007.12.014

[9] Boyd HA, Poulsen G, Wohlfahrt J, Murray JC, Feenstra B, Melbye M. Maternal contributions to preterm delivery. American journal of epidemiology. 2009;170(11):1358–64. 10.1093/aje/kwp324 19854807 PMC2800264

[10] Plunkett J, Feitosa MF, Trusgnich M, Wangler MF, Palomar L, Kistka ZA, et al Mother's genome or maternally-inherited genes acting in the fetus influence gestational age in familial preterm birth. Human heredity. 2009;68(3):209–19. 10.1159/000224641 19521103 PMC2869074

[11] York TP, Eaves LJ, Lichtenstein P, Neale MC, Svensson A, Latendresse S, et al Fetal and maternal genes' influence on gestational age in a quantitative genetic analysis of 244,000 Swedish births. American journal of epidemiology. 2013;178(4):543–50. 10.1093/aje/kwt005 23568591 PMC3736752

[12] Monangi NK, Brockway HM, House M, Zhang G, Muglia LJ. The genetics of preterm birth: Progress and promise. Seminars in perinatology. 2015;39(8):574–83. 10.1053/j.semperi.2015.09.005 26459968

[13] Strauss JF 3rd, Romero R, Gomez-Lopez N, Haymond-Thornburg H, Modi BP, Teves ME, et al Spontaneous preterm birth: advances toward the discovery of genetic predisposition. American journal of obstetrics and gynecology. 2018;218(3):294–314.e2. 10.1016/j.ajog.2017.12.009 29248470 PMC5834399

[14] Zhang G, Feenstra B, Bacelis J, Liu X, Muglia LM, Juodakis J, et al Genetic Associations with Gestational Duration and Spontaneous Preterm Birth. The New England journal of medicine. 2017;377(12):1156–67. 10.1056/NEJMoa1612665 28877031 PMC5561422

[15] Wang Z, Liu X, Yang BZ, Gelernter J. The role and challenges of exome sequencing in studies of human diseases. Frontiers in genetics. 2013;4:160 10.3389/fgene.2013.00160 24032039 PMC3752524

[16] Bamshad MJ, Ng SB, Bigham AW, Tabor HK, Emond MJ, Nickerson DA, et al Exome sequencing as a tool for Mendelian disease gene discovery. Nature reviews Genetics. 2011;12(11):745–55. 10.1038/nrg3031 21946919

[17] Cirulli ET, Goldstein DB. Uncovering the roles of rare variants in common disease through whole-genome sequencing. Nature reviews Genetics. 2010;11(6):415–25. 10.1038/nrg2779 20479773

[18] Uzun A, Schuster J, McGonnigal B, Schorl C, Dewan A, Padbury J. Targeted Sequencing and Meta-Analysis of Preterm Birth. PloS one. 2016;11(5):e0155021 10.1371/journal.pone.0155021 27163930 PMC4862658

[19] Modi BP, Teves ME, Pearson LN, Parikh HI, Haymond-Thornburg H, Tucker JL, et al Mutations in fetal genes involved in innate immunity and host defense against microbes increase risk of preterm premature rupture of membranes (PPROM). Molecular genetics & genomic medicine. 2017;5(6):720–9. 29178652 10.1002/mgg3.330 PMC5702565

[20] Modi BP, Teves ME, Pearson LN, Parikh HI, Chaemsaithong P, Sheth NU, et al Rare mutations and potentially damaging missense variants in genes encoding fibrillar collagens and proteins involved in their production are candidates for risk for preterm premature rupture of membranes. PloS one. 2017;12(3):e0174356 10.1371/journal.pone.0174356 28346524 PMC5367779

[21] Richards S, Aziz N, Bale S, Bick D, Das S, Gastier-Foster J, et al Standards and guidelines for the interpretation of sequence variants: a joint consensus recommendation of the American College of Medical Genetics and Genomics and the Association for Molecular Pathology. Genetics in medicine: official journal of the American College of Medical Genetics. 2015;17(5):405–24. 25741868 10.1038/gim.2015.30 PMC4544753

[22] Nishi H, Hashimoto K, Panchenko AR. Phosphorylation in protein-protein binding: effect on stability and function. Structure (London, England: 1993). 2011;19(12):1807–15. 10.1016/j.str.2011.09.021 PMC3240861 22153503

[23] Fourie AM, Peterson PA, Yang Y. Characterization and regulation of the major histocompatibility complex-encoded proteins Hsp70-Hom and Hsp70-1/2. Cell stress & chaperones. 2001;6(3):282–95. 11599570 10.1379/1466-1268(2001)006<0282:carotm>2.0.co;2 PMC434410

[24] Daugaard M, Rohde M, Jaattela M. The heat shock protein 70 family: Highly homologous proteins with overlapping and distinct functions. FEBS letters. 2007;581(19):3702–10. 10.1016/j.febslet.2007.05.039 17544402

[25] Hartl FU. Molecular chaperones in cellular protein folding. Nature. 1996;381(6583):571–80. 10.1038/381571a0 8637592

[26] Schlesinger MJ. Heat shock proteins. The Journal of biological chemistry. 1990;265(21):12111–4. 2197269

[27] Hartl FU, Bracher A, Hayer-Hartl M. Molecular chaperones in protein folding and proteostasis. Nature. 2011;475(7356):324–32. 10.1038/nature10317 21776078

[28] Molvarec A, Tamasi L, Losonczy G, Madach K, Prohaszka Z, Rigo J Jr. Circulating heat shock protein 70 (HSPA1A) in normal and pathological pregnancies. Cell stress & chaperones. 2010;15(3):237–47. 19821156 10.1007/s12192-009-0146-5 PMC2866993

[29] Chaiworapongsa T, Erez O, Kusanovic JP, Vaisbuch E, Mazaki-Tovi S, Gotsch F, et al Amniotic fluid heat shock protein 70 concentration in histologic chorioamnionitis, term and preterm parturition. The journal of maternal-fetal & neonatal medicine: the official journal of the European Association of Perinatal Medicine, the Federation of Asia and Oceania Perinatal Societies, the International Society of Perinatal Obstet. 2008;21(7):449–61. 10.1080/14767050802054550 PMC2517420 18570125

[30] Fekete A, Ver A, Bogi K, Treszl A, Rigo J Jr. Is preeclampsia associated with higher frequency of HSP70 gene polymorphisms? European journal of obstetrics, gynecology, and reproductive biology. 2006;126(2):197–200. 10.1016/j.ejogrb.2005.08.021 16202503

[31] Brocchieri L, Conway de Macario E, Macario AJ. hsp70 genes in the human genome: Conservation and differentiation patterns predict a wide array of overlapping and specialized functions. BMC evolutionary biology. 2008;8:19 10.1186/1471-2148-8-19 18215318 PMC2266713

[32] Zhang P, Leu JI, Murphy ME, George DL, Marmorstein R. Crystal structure of the stress-inducible human heat shock protein 70 substrate-binding domain in complex with peptide substrate. PloS one. 2014;9(7):e103518 10.1371/journal.pone.0103518 25058147 PMC4110032

[33] Takahashi S, Andreoletti G, Chen R, Munehira Y, Batra A, Afzal NA, et al De novo and rare mutations in the HSPA1L heat shock gene associated with inflammatory bowel disease. Genome medicine. 2017;9(1):8 10.1186/s13073-016-0394-9 28126021 PMC5270254

[34] Getahun D, Fassett MJ, Longstreth GF, Koebnick C, Langer-Gould AM, Strickland D, et al Association between maternal inflammatory bowel disease and adverse perinatal outcomes. Journal of perinatology: official journal of the California Perinatal Association. 2014;34(6):435–40. 24651735 10.1038/jp.2014.41

[35] Velez DR, Fortunato S, Thorsen P, Lombardi SJ, Williams SM, Menon R. Spontaneous preterm birth in African Americans is associated with infection and inflammatory response gene variants. American journal of obstetrics and gynecology. 2009;200(2):209.e1–27. 19019335 10.1016/j.ajog.2008.08.051 PMC4829203

[36] Capece A, Vasieva O, Meher S, Alfirevic Z, Alfirevic A. Pathway analysis of genetic factors associated with spontaneous preterm birth and pre-labor preterm rupture of membranes. PloS one. 2014;9(9):e108578 10.1371/journal.pone.0108578 25264875 PMC4181300

[37] Nollen EA, Morimoto RI. Chaperoning signaling pathways: molecular chaperones as stress-sensing 'heat shock' proteins. Journal of cell science. 2002;115(Pt 14):2809–16. 12082142 10.1242/jcs.115.14.2809

[38] Schoneveld OJ, Gaemers IC, Lamers WH. Mechanisms of glucocorticoid signalling. Biochimica et biophysica acta. 2004;1680(2):114–28. 10.1016/j.bbaexp.2004.09.004 15488991

[39] Kadmiel M, Cidlowski JA. Glucocorticoid receptor signaling in health and disease. Trends in pharmacological sciences. 2013;34(9):518–30. 10.1016/j.tips.2013.07.003 23953592 PMC3951203

[40] Engler JB, Kursawe N, Solano ME, Patas K, Wehrmann S, Heckmann N, et al Glucocorticoid receptor in T cells mediates protection from autoimmunity in pregnancy. Proceedings of the National Academy of Sciences of the United States of America. 2017;114(2):E181–e90. 10.1073/pnas.1617115114 28049829 PMC5240705

[41] Lei K, Chen L, Georgiou EX, Sooranna SR, Khanjani S, Brosens JJ, et al Progesterone acts via the nuclear glucocorticoid receptor to suppress IL-1beta-induced COX-2 expression in human term myometrial cells. PloS one. 2012;7(11):e50167 10.1371/journal.pone.0050167 23209664 PMC3509141

[42] Menon R. Spontaneous preterm birth, a clinical dilemma: etiologic, pathophysiologic and genetic heterogeneities and racial disparity. Acta obstetricia et gynecologica Scandinavica. 2008;87(6):590–600. 10.1080/00016340802005126 18568457

[43] Gomez-Lopez N, StLouis D, Lehr MA, Sanchez-Rodriguez EN, Arenas-Hernandez M. Immune cells in term and preterm labor. Cellular & molecular immunology. 2014;11(6):571–81. 24954221 10.1038/cmi.2014.46 PMC4220837

[44] Haataja R, Karjalainen MK, Luukkonen A, Teramo K, Puttonen H, Ojaniemi M, et al Mapping a new spontaneous preterm birth susceptibility gene, IGF1R, using linkage, haplotype sharing, and association analysis. PLoS genetics. 2011;7(2):e1001293 10.1371/journal.pgen.1001293 21304894 PMC3033387

[45] Karjalainen MK, Huusko JM, Ulvila J, Sotkasiira J, Luukkonen A, Teramo K, et al A potential novel spontaneous preterm birth gene, AR, identified by linkage and association analysis of X chromosomal markers. PloS one. 2012;7(12):e51378 10.1371/journal.pone.0051378 23227263 PMC3515491

[46] Varilo T. The age of the mutations in the Finnish disease heritage; a genealogical and linkage disequilibrium study. [PhD thesis]. University of Helsinki, Department of Medical Genetics, Faculty of Medicine and Department of Human Molecular Genetics, National Public Health Institute, Helsinki.1999.

[47] Soden SE, Saunders CJ, Willig LK, Farrow EG, Smith LD, Petrikin JE, et al Effectiveness of exome and genome sequencing guided by acuity of illness for diagnosis of neurodevelopmental disorders. Science translational medicine. 2014;6(265):265ra168 10.1126/scitranslmed.3010076 25473036 PMC4286868

[48] Li H, Durbin R. Fast and accurate short read alignment with Burrows-Wheeler transform. Bioinformatics (Oxford, England). 2009;25(14):1754–60. 10.1093/bioinformatics/btp324 PMC2705234 19451168

[49] McKenna A, Hanna M, Banks E, Sivachenko A, Cibulskis K, Kernytsky A, et al The Genome Analysis Toolkit: a MapReduce framework for analyzing next-generation DNA sequencing data. Genome research. 2010;20(9):1297–303. 10.1101/gr.107524.110 20644199 PMC2928508

[50] Saunders CJ, Miller NA, Soden SE, Dinwiddie DL, Noll A, Alnadi NA, et al Rapid whole-genome sequencing for genetic disease diagnosis in neonatal intensive care units. Science translational medicine. 2012;4(154):154ra35. 10.1126/scitranslmed.3004041 PMC4283791 23035047

[51] Zhang G, Bacelis J, Lengyel C, Teramo K, Hallman M, Helgeland O, et al Assessing the Causal Relationship of Maternal Height on Birth Size and Gestational Age at Birth: A Mendelian Randomization Analysis. PLoS medicine. 2015;12(8):e1001865 10.1371/journal.pmed.1001865 26284790 PMC4540580

[52] Delaneau O, Zagury JF, Marchini J. Improved whole-chromosome phasing for disease and population genetic studies. Nature methods. 2013;10(1):5–6. 10.1038/nmeth.2307 23269371

[53] Howie BN, Donnelly P, Marchini J. A flexible and accurate genotype imputation method for the next generation of genome-wide association studies. PLoS genetics. 2009;5(6):e1000529 10.1371/journal.pgen.1000529 19543373 PMC2689936

[54] Marchini J, Howie B, Myers S, McVean G, Donnelly P. A new multipoint method for genome-wide association studies by imputation of genotypes. Nature genetics. 2007;39(7):906–13. 10.1038/ng2088 17572673

[55] Kircher M, Witten DM, Jain P, O'Roak BJ, Cooper GM, Shendure J. A general framework for estimating the relative pathogenicity of human genetic variants. Nature genetics. 2014;46(3):310–5. 10.1038/ng.2892 24487276 PMC3992975

[56] Blom N, Sicheritz-Ponten T, Gupta R, Gammeltoft S, Brunak S. Prediction of post-translational glycosylation and phosphorylation of proteins from the amino acid sequence. Proteomics. 2004;4(6):1633–49. 10.1002/pmic.200300771 15174133

[57] Pettersen EF, Goddard TD, Huang CC, Couch GS, Greenblatt DM, Meng EC, et al UCSF Chimera—a visualization system for exploratory research and analysis. Journal of computational chemistry. 2004;25(13):1605–12. 10.1002/jcc.20084 15264254

