# Correction: Whole exome sequencing reveals *HSPA1L* as a genetic risk factor for spontaneous preterm birth

**DOI:** 10.1371/journal.pgen.1007673

**Published:** 2018-09-13

**Authors:** Johanna M. Huusko, Minna K. Karjalainen, Britney E. Graham, Ge Zhang, Emily G. Farrow, Neil A. Miller, Bo Jacobsson, Haley R. Eidem, Jeffrey C. Murray, Bruce Bedell, Patrick Breheny, Noah W. Brown, Frans L. Bødker, Nadia K. Litterman, Pan-Pan Jiang, Laura Russell, David A. Hinds, Youna Hu, the 23andMe Research Team, Antonis Rokas, Kari Teramo, Kaare Christensen, Scott M. Williams, Mika Rämet, Stephen F. Kingsmore, Kelli K. Ryckman, Mikko Hallman, Louis J. Muglia

The Data Availability statement for this paper contains inactive web links. The updated statement with active web links is as follows:

For the Discovery whole exome data, the requests of the data underlying the findings reported in this study of the families from northern Finland (Oulu), as well as individual-level phenotype and SNP genotype data from the Finnish (Helsinki) birth cohort (validation GWAS dataset), are available through the March of Dimes Prematurity Research Center Ohio Collaborative (https://www.marchofdimes.org/research/ohio-collaborative-overview.aspx) and access will be approved by the Leadership Committee through its director of operations, Joanne Chappell (joanne.chappell@cchmc.org). Some restrictions may apply for the protection of privacy. Any use of the Danish Replication WES data must be approved by the Danish National IRB and the Danish Data Protection Agency through an application facilitated by the Danish Principal Investigator (kchristensen@health.sdu.dk) at the University of Southern Denmark. Some restrictions may apply for the protection of privacy.

For the validation GWAS datasets: the summary statistical outcomes of the top 10,000 SNPs for the 23andMe data have been deposited in the GeneStation repository (www.genestation.org/analysis/gwas/Zhang_2017/discovery), and summary statistics of the complete data set are available on request from 23andMe. Access to the DNBC individual-level data can be obtained through dbGaP Authorized Access portal (https://dbgap.ncbi.nlm.nih.gov/dbgap/aa). The Norwegian cohort data underlying this study was obtained from a third party and is a subject to some legal restrictions. The data originates from the Norwegian Mother Child cohort (MoBa), which is controlled by the MoBa Scientific Management Group. The research data can be accessed via electronic application forms at https://www.fhi.no/en/studies/moba/. Data can be requested of all interested researchers qualifying for the requirements established by the MoBa Scientific Management Group. For more information please contact datatilgang@fhi.no.
