# Supplementary material for: Whole exome sequencing reveals HSPA1L as a genetic risk factor for spontaneous preterm birth
Source: PLoS Genet. 2018 Jul 12;14(7):e1007394. doi: 10.1371/journal.pgen.1007394 (PMC6042692; doi:10.1371/journal.pgen.1007394)
Supplement: S1 Fig — Circles represent females and squares males; symbols with a line intersecting indicate that individual is deceased. Diamonds denote an unspecified number of infants born at term, i.e. gestational age (GA) ≥37 weeks. Letters above symbols indicate individuals for whom WES data was gathered and an asterisk next to the letter denotes the individual as a recurrent mother. Letters inside brackets indicate grandmothers with term, twin or non-spontaneous deliveries, and were not included in the primary analyses. Pedigrees were created using Progeny Pedigree tool (https://pedigree.progenygenetics.com/). (PDF) [file pgen.1007394.s001.pdf]

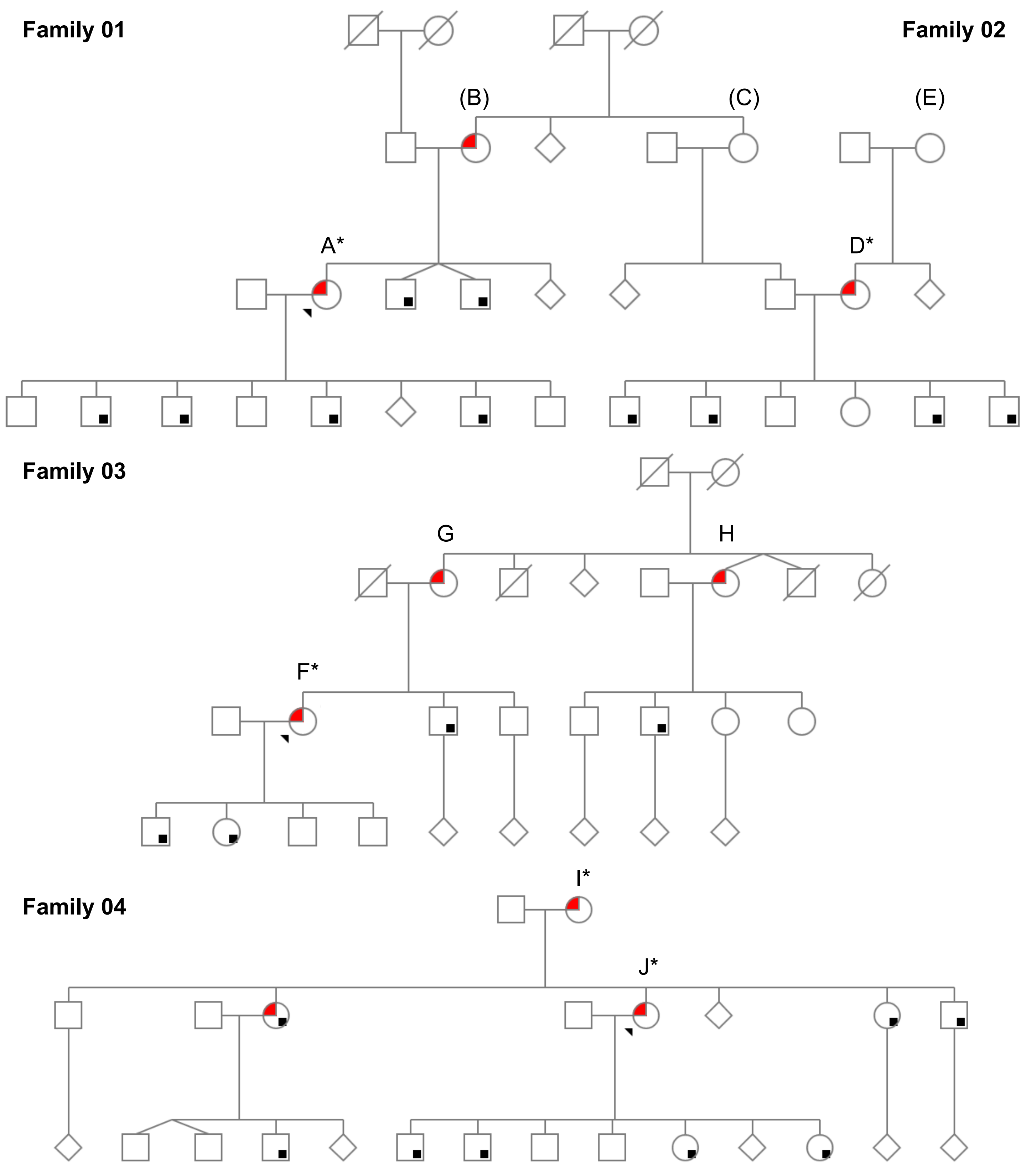
Family 01
Family 02
(B)
(C)
(E)
A*
D*
Family 03
G
H
F*
Family 04
I*
J*

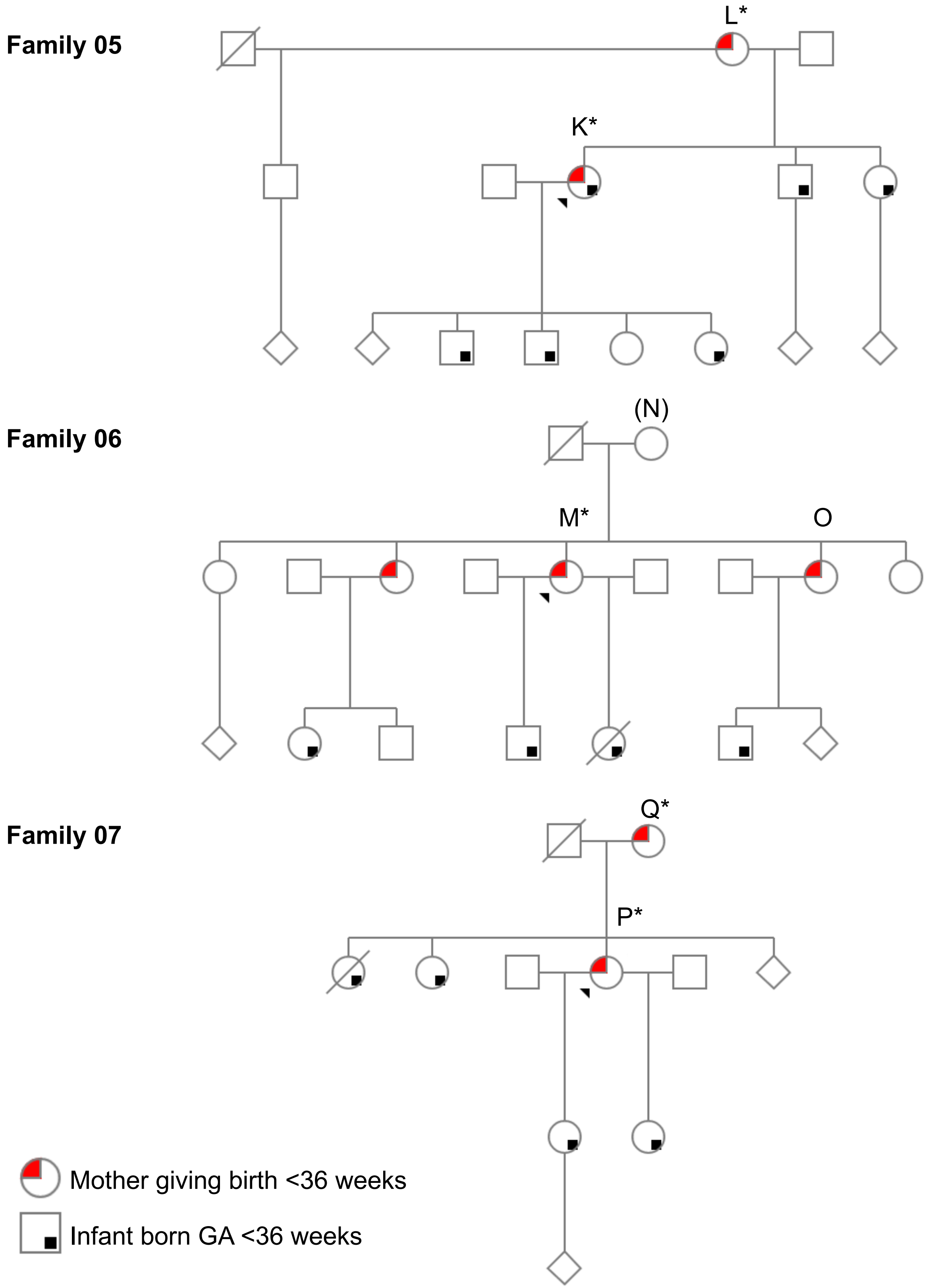

Family 05
L*
K*
Family 06
(N)
M*
O
Family 07
Q*
P*
Mother giving birth <36 weeks
Infant born GA <36 weeks
