# Supplementary figures and images for: Whole exome sequencing reveals HSPA1L as a genetic risk factor for spontaneous preterm birth
Source: PLoS Genet. 2018 Jul 12;14(7):e1007394. doi: 10.1371/journal.pgen.1007394 (PMC6042692; doi:10.1371/journal.pgen.1007394)

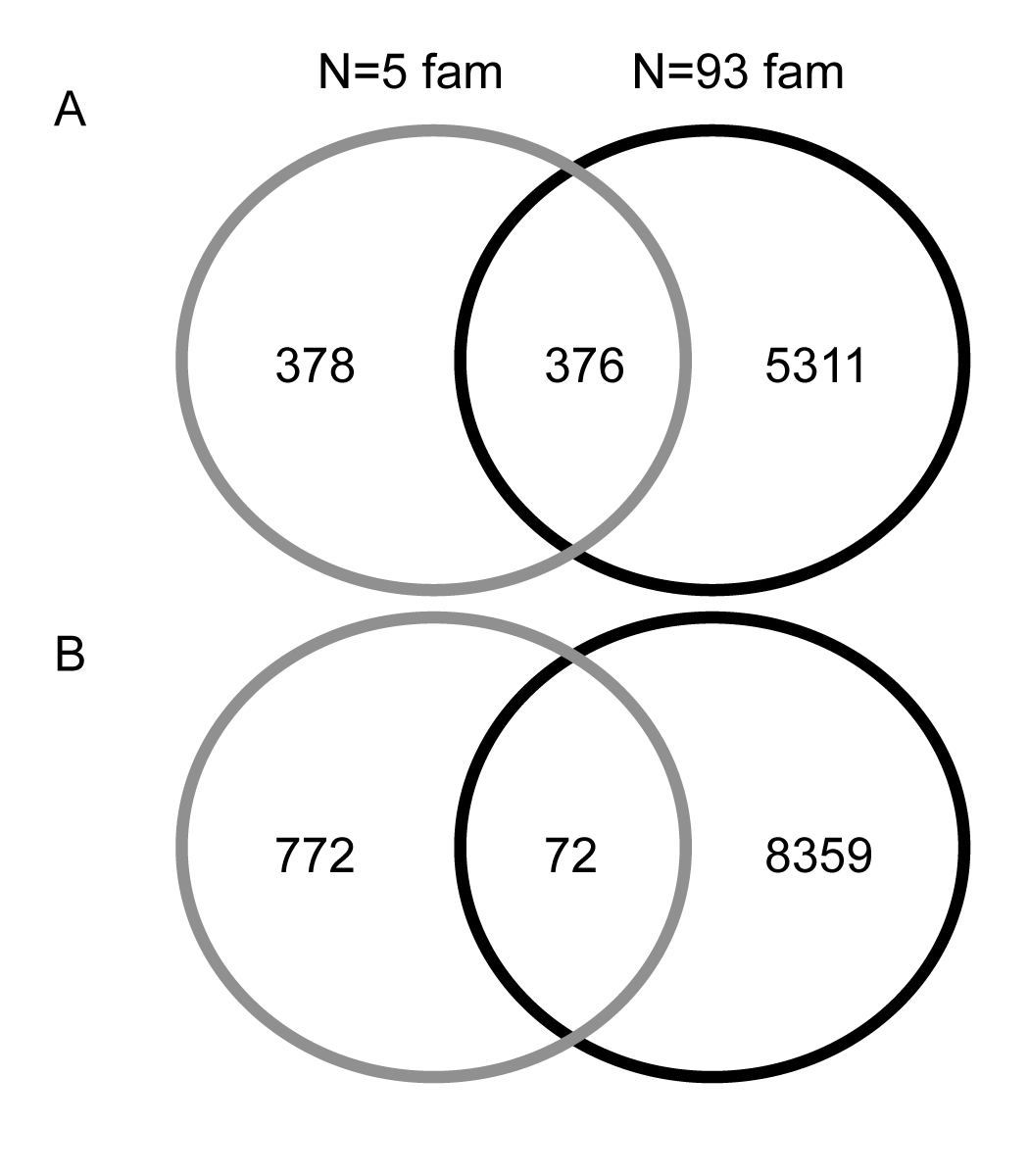

Supplement: S2 Fig — For both the Discovery (total of 5 families) and Replication (total of 93 families) populations, these analyses only included rare variants that passed the filters of multiple annotating software tools and were shared by affected family members. The total number of genes (A) and variants (B) that were common between the two populations are presented in the middle of the Venn diagram. (TIF) [file pgen.1007394.s002.tif]

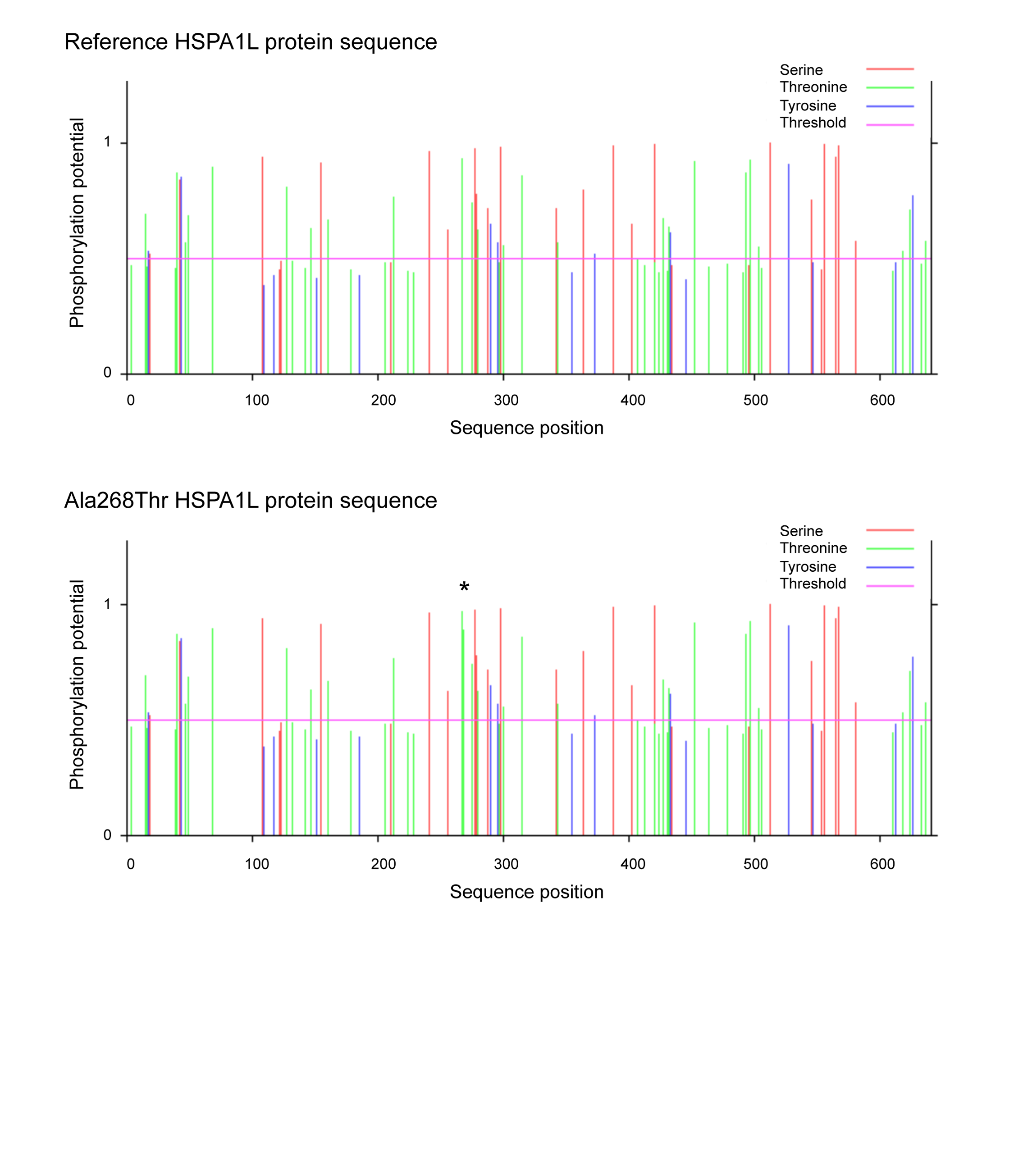

Supplement: S3 Fig — The potential effect of Ala268Thr on HSPA1L protein sequence was predicted using NetPhos 3.1. This missense variant generates an additional phosphorylation site on the HSPA1L sequence with high confidence (0.855; above the threshold of 0.5). The T268-p phosphorylation site is marked with an asterisk. (TIF) [file pgen.1007394.s003.tif]

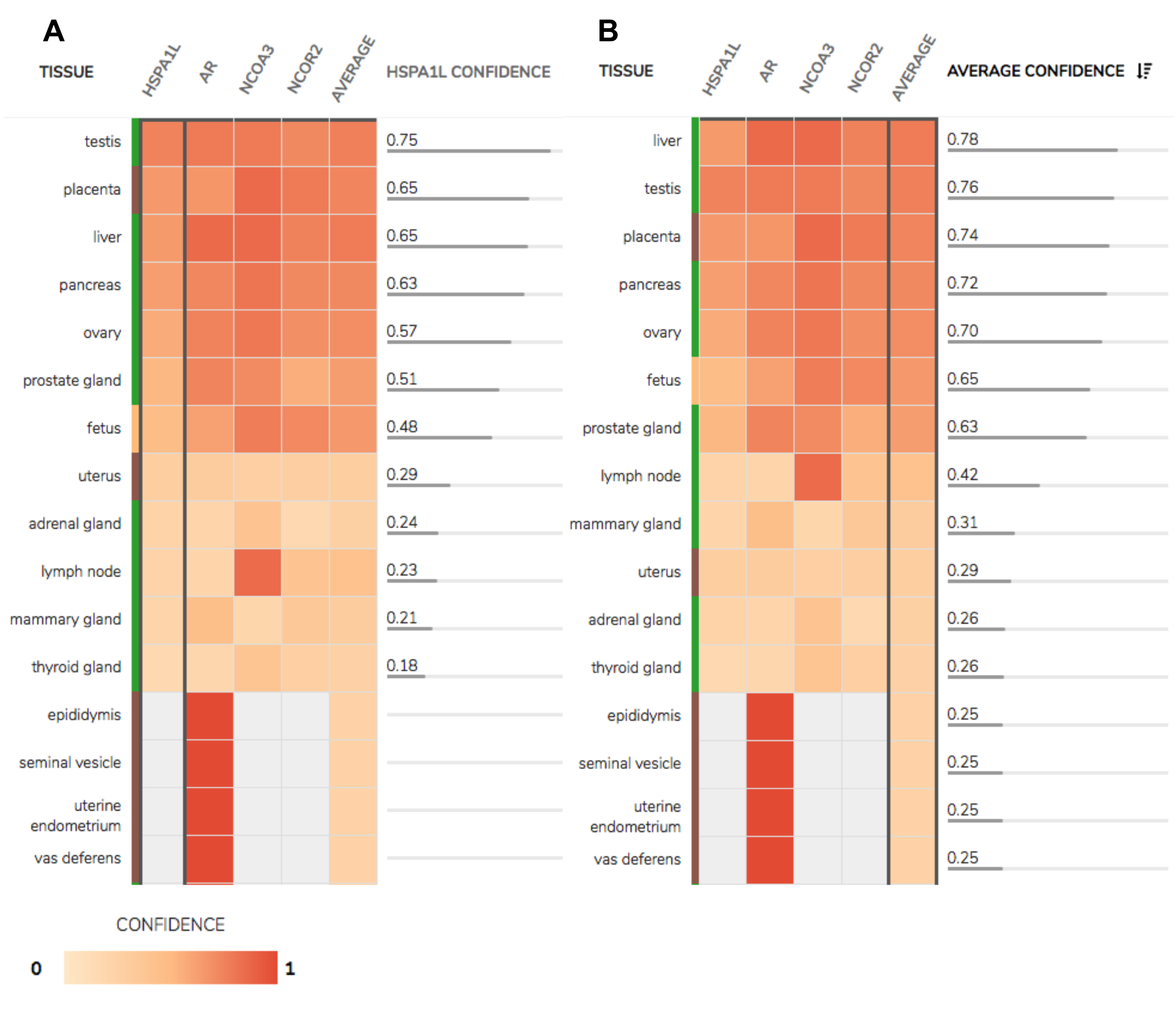

Supplement: S4 Fig — (A) HSPA1L is expressed with reasonable confidence in placental tissue (0.65), ovarian (0.57) and fetal tissues (0.48) as well as in uterus (0.29). (B) For HSPA1L, AR, NCOA3 and NCOR2 together, the average expression confidence is high in placenta (0.74), ovary (0.70), fetus (0.65), and moderate in uterus (0.29), indicating high confidence for expression in female reproductive system overall. Tissue expression was established using HumanBase (http://hb.flatironinstitute.org). (TIF) [file pgen.1007394.s004.tif]
