# Supplementary material for: Whole exome sequencing reveals HSPA1L as a genetic risk factor for spontaneous preterm birth
Source: PLoS Genet. 2018 Jul 12;14(7):e1007394. doi: 10.1371/journal.pgen.1007394 (PMC6042692; doi:10.1371/journal.pgen.1007394)
Supplement: S1 Table — (DOCX) [file pgen.1007394.s005.docx]

**S1 Table. Pathway results for Finnish mothers with recurrent preterm births (n=10).**

| **Rank** | **Pathway name** | **P-value** | **No. of Genes** | **No. of Variants** | **No. of Cases** |
| --- | --- | --- | --- | --- | --- |
| 1 | Glucocorticoid Receptor Signaling | 1.673E-8 | 13 | 36 | 10 |
| 2 | Estrogen Receptor Signaling | 5.067E-7 | 8 | 27 | 10 |
| 3 | AMPK Signaling | 9.804E-7 | 10 | 28 | 10 |
| 4 | TR/RXR Activation | 1.310E-6 | 7 | 15 | 10 |
| 5 | Breast Cancer Regulation by Stathmin1 | 1.389E-6 | 9 | 27 | 10 |
| 6 | Antigen Presentation Pathway | 2.661E-6 | 5 | 71 | 10 |
| 7 | Huntington's Disease Signaling | 4.384E-6 | 10 | 20 | 10 |
| 8 | phagosome maturation | 4.456E-6 | 7 | 65 | 10 |
| 9 | Axonal Guidance Signaling | 1.489E-5 | 12 | 29 | 10 |
| 10 | Integrin Signaling | 2.218E-5 | 8 | 31 | 10 |
| 11 | FAK Signaling | 6.009E-5 | 6 | 13 | 10 |
| 12 | Actin Cytoskeleton Signaling | 9.271E-5 | 8 | 33 | 10 |
| 13 | Amyotrophic Lateral Sclerosis Signaling | 1.027E-4 | 6 | 9 | 10 |
| 14 | Notch Signaling | 1.080E-4 | 4 | 13 | 9 |
| 15 | Thrombin Signaling | 1.132E-4 | 7 | 17 | 10 |
| 16 | Cdc42 Signaling | 1.311E-4 | 8 | 81 | 10 |
| 17 | Epithelial Adherens Junction Signaling | 1.470E-4 | 6 | 18 | 10 |
| 18 | Aldosterone Signaling in Epithelial Cells | 1.823E-4 | 6 | 14 | 9 |
| 19 | NRF2-mediated Oxidative Stress Response | 3.625E-4 | 7 | 22 | 10 |
| 20 | Germ Cell-Sertoli Cell Junction Signaling | 3.659E-4 | 6 | 16 | 10 |
| 21 | Androgen Signaling | 5.567E-4 | 6 | 27 | 10 |
| 22 | Xenobiotic Metabolism Signaling | 8.109E-4 | 7 | 18 | 10 |
| 23 | Molecular Mechanisms of Cancer | 8.127E-4 | 9 | 21 | 10 |
| 24 | Sertoli Cell-Sertoli Cell Junction Signaling | 9.287E-4 | 6 | 17 | 10 |
| 25 | Reelin Signaling in Neurons | 9.399E-4 | 4 | 7 | 10 |
| 26 | RAR Activation | 9.484E-4 | 6 | 10 | 10 |
| 27 | Ceramide Signaling | 1.362E-3 | 4 | 13 | 10 |
| 28 | autophagy | 1.616E-3 | 3 | 5 | 10 |
| 29 | eNOS Signaling | 1.683E-3 | 5 | 10 | 10 |
| 30 | OX40 Signaling Pathway | 1.763E-3 | 5 | 71 | 10 |
| 31 | Allograft Rejection Signaling | 1.888E-3 | 5 | 71 | 10 |
| 32 | PKCÎ¸ Signaling in T Lymphocytes | 1.999E-3 | 6 | 36 | 10 |
| 33 | Cleavage and Polyadenylation of Pre-mRNA | 2.050E-3 | 2 | 7 | 8 |
| 34 | Non-Small Cell Lung Cancer Signaling | 2.154E-3 | 4 | 11 | 9 |
| 35 | Glioblastoma Multiforme Signaling | 2.205E-3 | 5 | 17 | 10 |
| 36 | nNOS Signaling in Neurons | 2.226E-3 | 3 | 5 | 10 |
| 37 | Gustation Pathway | 2.303E-3 | 5 | 18 | 10 |
| 38 | Tight Junction Signaling | 2.895E-3 | 5 | 14 | 8 |
| 39 | GNRH Signaling | 2.954E-3 | 5 | 15 | 10 |
| 40 | Crosstalk between Dendritic Cells and Natural Killer Cells | 3.131E-3 | 4 | 61 | 10 |
| 41 | Colorectal Cancer Metastasis Signaling | 3.175E-3 | 6 | 14 | 10 |
| 42 | EIF2 Signaling | 3.198E-3 | 5 | 52 | 10 |
| 43 | Chronic Myeloid Leukemia Signaling | 3.217E-3 | 4 | 17 | 10 |
| 44 | Paxillin Signaling | 3.393E-3 | 4 | 11 | 10 |
| 45 | 14-3-3-mediated Signaling | 3.393E-3 | 4 | 9 | 10 |
| 46 | mTOR Signaling | 4.167E-3 | 5 | 13 | 10 |
| 47 | Type II Diabetes Mellitus Signaling | 4.167E-3 | 5 | 12 | 10 |
| 48 | NGF Signaling | 4.374E-3 | 4 | 9 | 10 |
| 49 | ILK Signaling | 4.559E-3 | 5 | 22 | 10 |
| 50 | ERK5 Signaling | 4.839E-3 | 3 | 9 | 10 |
| 51 | PAK Signaling | 5.164E-3 | 4 | 13 | 10 |
| 52 | Renin-Angiotensin Signaling | 5.406E-3 | 4 | 11 | 10 |
| 53 | EGF Signaling | 5.592E-3 | 3 | 7 | 8 |
| 54 | LXR/RXR Activation | 5.656E-3 | 4 | 8 | 9 |
| 55 | Protein Kinase A Signaling | 6.081E-3 | 7 | 53 | 10 |
| 56 | CREB Signaling in Neurons | 6.287E-3 | 5 | 15 | 10 |
| 57 | CCR3 Signaling in Eosinophils | 6.450E-3 | 4 | 13 | 10 |
| 58 | Basal Cell Carcinoma Signaling | 7.533E-3 | 3 | 5 | 8 |
| 59 | Role of NFAT in Cardiac Hypertrophy | 7.590E-3 | 5 | 15 | 10 |
| 60 | IL-4 Signaling | 8.508E-3 | 3 | 26 | 9 |
| 61 | VDR/RXR Activation | 9.285E-3 | 3 | 5 | 10 |
| 62 | PEDF Signaling | 9.553E-3 | 3 | 6 | 7 |
| 63 | Calcium Signaling | 9.741E-3 | 5 | 15 | 10 |
| 64 | Renal Cell Carcinoma Signaling | 9.825E-3 | 3 | 11 | 10 |
