# Supplementary material for: Whole exome sequencing reveals HSPA1L as a genetic risk factor for spontaneous preterm birth
Source: PLoS Genet. 2018 Jul 12;14(7):e1007394. doi: 10.1371/journal.pgen.1007394 (PMC6042692; doi:10.1371/journal.pgen.1007394)
Supplement: S2 Table — (DOCX) [file pgen.1007394.s006.docx]

**S2 Table. Pathway results for Finnish families (n=5) with multiple affected individuals: only pathways seen in at least two different families are listed.**

| **Family ID** | **Pathway Name** | **P-value** | **No. of Families** | **No. of Genes** | **No. of Variants** |
| --- | --- | --- | --- | --- | --- |
| 03 | Glucocorticoid Receptor Signaling | 5.89E-07 | 5 | 11 | 11 |
| 04 | Glucocorticoid Receptor Signaling | 6.66E-07 | 5 | 9 | 9 |
| 05 | Glucocorticoid Receptor Signaling | 2.96E-04 | 5 | 6 | 7 |
| 06 | Glucocorticoid Receptor Signaling | 5.61E-03 | 5 | 4 | 4 |
| 07 | Glucocorticoid Receptor Signaling | 1.62E-03 | 5 | 5 | 5 |
| 03 | Sertoli Cell-Sertoli Cell Junction Signaling | 9.39E-05 | 4 | 7 | 7 |
| 04 | Sertoli Cell-Sertoli Cell Junction Signaling | 4.19E-03 | 4 | 4 | 5 |
| 05 | Sertoli Cell-Sertoli Cell Junction Signaling | 3.16E-03 | 4 | 4 | 6 |
| 07 | Sertoli Cell-Sertoli Cell Junction Signaling | 2.54E-03 | 4 | 4 | 5 |
| 03 | Actin Cytoskeleton Signaling | 8.21E-06 | 3 | 9 | 10 |
| 05 | Actin Cytoskeleton Signaling | 7.14E-03 | 3 | 4 | 4 |
| 06 | Actin Cytoskeleton Signaling | 3.29E-05 | 3 | 6 | 7 |
| 03 | Aldosterone Signaling in Epithelial Cells | 7.46E-03 | 3 | 4 | 4 |
| 04 | Aldosterone Signaling in Epithelial Cells | 1.38E-03 | 3 | 4 | 4 |
| 06 | Aldosterone Signaling in Epithelial Cells | 5.20E-03 | 3 | 3 | 3 |
| 03 | Estrogen Receptor Signaling | 4.92E-05 | 3 | 6 | 7 |
| 04 | Estrogen Receptor Signaling | 7.21E-04 | 3 | 4 | 4 |
| 06 | Estrogen Receptor Signaling | 3.19E-03 | 3 | 3 | 3 |
| 03 | Hepatic Cholestasis | 4.02E-03 | 3 | 5 | 6 |
| 04 | Hepatic Cholestasis | 4.13E-03 | 3 | 4 | 4 |
| 07 | Hepatic Cholestasis | 2.50E-03 | 3 | 4 | 5 |
| 03 | HGF Signaling | 7.91E-04 | 3 | 5 | 5 |
| 05 | HGF Signaling | 8.22E-03 | 3 | 3 | 4 |
| 06 | HGF Signaling | 4.30E-03 | 3 | 3 | 4 |
| 03 | Integrin Signaling | 1.57E-06 | 3 | 9 | 10 |
| 05 | Integrin Signaling | 4.08E-04 | 3 | 5 | 5 |
| 06 | Integrin Signaling | 1.49E-03 | 3 | 4 | 5 |
| 03 | Protein Kinase A Signaling | 4.09E-05 | 3 | 10 | 11 |
| 04 | Protein Kinase A Signaling | 5.50E-06 | 3 | 9 | 9 |
| 07 | Protein Kinase A Signaling | 4.88E-03 | 3 | 5 | 5 |
| 03 | Xenobiotic Metabolism Signaling | 2.11E-07 | 3 | 11 | 13 |
| 05 | Xenobiotic Metabolism Signaling | 8.85E-03 | 3 | 4 | 5 |
| 07 | Xenobiotic Metabolism Signaling | 7.17E-03 | 3 | 4 | 5 |
| 04 | 14-3-3-mediated Signaling | 4.74E-04 | 2 | 4 | 5 |
| 07 | 14-3-3-mediated Signaling | 3.80E-03 | 2 | 3 | 3 |
| 03 | AMPK Signaling | 5.53E-07 | 2 | 10 | 11 |
| 07 | AMPK Signaling | 4.57E-03 | 2 | 4 | 4 |
| 03 | Androgen Signaling | 3.99E-04 | 2 | 6 | 6 |
| 04 | Androgen Signaling | 3.28E-04 | 2 | 5 | 5 |
| 04 | April Mediated Signaling | 4.28E-04 | 2 | 3 | 3 |
| 07 | April Mediated Signaling | 7.15E-03 | 2 | 2 | 2 |
| 03 | Axonal Guidance Signaling | 4.14E-05 | 2 | 11 | 11 |
| 04 | Axonal Guidance Signaling | 8.95E-04 | 2 | 7 | 8 |
| 03 | Breast Cancer Regulation by Stathmin1 | 5.15E-04 | 2 | 6 | 7 |
| 04 | Breast Cancer Regulation by Stathmin1 | 3.96E-05 | 2 | 6 | 8 |
| 03 | Cardiac Hypertrophy Signaling | 9.16E-03 | 2 | 5 | 5 |
| 06 | Cardiac Hypertrophy Signaling | 2.74E-03 | 2 | 4 | 5 |
| 03 | Caveolar-mediated Endocytosis Signaling | 3.50E-06 | 2 | 6 | 7 |
| 05 | Caveolar-mediated Endocytosis Signaling | 1.67E-03 | 2 | 3 | 4 |
| 03 | CCR3 Signaling in Eosinophils | 5.17E-03 | 2 | 4 | 4 |
| 04 | CCR3 Signaling in Eosinophils | 9.22E-03 | 2 | 3 | 3 |
| 06 | CD27 Signaling in Lymphocytes | 8.83E-03 | 2 | 2 | 3 |
| 07 | CD27 Signaling in Lymphocytes | 6.53E-04 | 2 | 3 | 4 |
| 03 | CREB Signaling in Neurons | 1.54E-05 | 2 | 8 | 8 |
| 04 | CREB Signaling in Neurons | 4.82E-03 | 2 | 4 | 4 |
| 03 | Dopamine-DARPP32 Feedback in cAMP Signaling | 2.96E-03 | 2 | 5 | 6 |
| 04 | Dopamine-DARPP32 Feedback in cAMP Signaling | 3.19E-03 | 2 | 4 | 4 |
| 04 | EIF2 Signaling | 2.72E-03 | 2 | 4 | 7 |
| 06 | EIF2 Signaling | 8.62E-04 | 2 | 4 | 4 |
| 03 | Ephrin Receptor Signaling | 1.56E-04 | 2 | 7 | 7 |
| 05 | Ephrin Receptor Signaling | 4.23E-03 | 2 | 4 | 4 |
| 03 | ERK/MAPK Signaling | 8.92E-04 | 2 | 6 | 7 |
| 05 | ERK/MAPK Signaling | 3.85E-03 | 2 | 4 | 4 |
| 03 | FAK Signaling | 4.23E-05 | 2 | 6 | 6 |
| 05 | FAK Signaling | 4.85E-04 | 2 | 4 | 4 |
| 03 | Gap Junction Signaling | 1.79E-03 | 2 | 5 | 5 |
| 04 | Gap Junction Signaling | 2.12E-04 | 2 | 5 | 6 |
| 04 | Germ Cell-Sertoli Cell Junction Signaling | 2.22E-03 | 2 | 4 | 5 |
| 05 | Germ Cell-Sertoli Cell Junction Signaling | 1.66E-03 | 2 | 4 | 5 |
| 04 | GNRH Signaling | 2.54E-03 | 2 | 4 | 4 |
| 06 | GNRH Signaling | 8.25E-03 | 2 | 3 | 4 |
| 03 | Hereditary Breast Cancer Signaling | 1.08E-04 | 2 | 6 | 6 |
| 07 | Hereditary Breast Cancer Signaling | 7.65E-03 | 2 | 3 | 3 |
| 03 | Huntington's Disease Signaling | 1.20E-04 | 2 | 8 | 8 |
| 04 | Huntington's Disease Signaling | 3.89E-05 | 2 | 7 | 7 |
| 06 | NGF Signaling | 2.84E-03 | 2 | 3 | 4 |
| 07 | NGF Signaling | 4.63E-03 | 2 | 3 | 4 |
| 03 | Nucleotide Excision Repair Pathway | 6.62E-04 | 2 | 3 | 3 |
| 06 | Nucleotide Excision Repair Pathway | 2.73E-03 | 2 | 2 | 2 |
| 03 | PAK Signaling | 4.40E-06 | 2 | 7 | 7 |
| 05 | PAK Signaling | 6.25E-03 | 2 | 3 | 3 |
| 03 | Paxillin Signaling | 2.93E-04 | 2 | 5 | 5 |
| 05 | Paxillin Signaling | 4.50E-03 | 2 | 3 | 3 |
| 04 | phagosome maturation | 6.56E-03 | 2 | 3 | 4 |
| 07 | phagosome maturation | 4.46E-03 | 2 | 3 | 4 |
| 03 | Phospholipase C Signaling | 8.91E-05 | 2 | 9 | 9 |
| 04 | Phospholipase C Signaling | 5.34E-03 | 2 | 5 | 6 |
| 03 | Protein Ubiquitination Pathway | 8.67E-03 | 2 | 5 | 7 |
| 04 | Protein Ubiquitination Pathway | 1.49E-05 | 2 | 7 | 7 |
| 03 | Rac Signaling | 9.02E-04 | 2 | 5 | 5 |
| 05 | Rac Signaling | 8.90E-03 | 2 | 3 | 3 |
| 03 | RAR Activation | 9.63E-05 | 2 | 7 | 7 |
| 07 | RAR Activation | 2.58E-03 | 2 | 4 | 4 |
| 03 | Regulation of Cellular Mechanics by Calpain Protease | 1.03E-04 | 2 | 5 | 5 |
| 05 | Regulation of Cellular Mechanics by Calpain Protease | 1.50E-04 | 2 | 4 | 4 |
| 03 | Renin-Angiotensin Signaling | 4.33E-03 | 2 | 4 | 4 |
| 04 | Renin-Angiotensin Signaling | 7.76E-04 | 2 | 4 | 4 |
| 03 | Role of NFAT in Cardiac Hypertrophy | 1.60E-04 | 2 | 7 | 8 |
| 04 | Role of NFAT in Cardiac Hypertrophy | 5.66E-03 | 2 | 4 | 4 |
| 03 | Thrombin Signaling | 3.55E-03 | 2 | 5 | 5 |
| 04 | Thrombin Signaling | 3.72E-03 | 2 | 4 | 5 |
| 03 | TR/RXR Activation | 1.63E-04 | 2 | 5 | 5 |
| 04 | TR/RXR Activation | 3.94E-03 | 2 | 3 | 3 |
