# Supplementary material for: Whole exome sequencing reveals HSPA1L as a genetic risk factor for spontaneous preterm birth
Source: PLoS Genet. 2018 Jul 12;14(7):e1007394. doi: 10.1371/journal.pgen.1007394 (PMC6042692; doi:10.1371/journal.pgen.1007394)
Supplement: S3 Table — (DOCX) [file pgen.1007394.s007.docx]

**S3 Table. Pathway results for Danish sister pairs (n=93): only pathways common for ≥20 families are listed.**

| **Rank** | **Pathway name** | **No. of Families** | **Average p-value** | **Median p-value** |
| --- | --- | --- | --- | --- |
| 1 | Protein Kinase A Signaling | 92 | 0.00038 | 0.000009 |
| 2 | Huntington's Disease Signaling | 87 | 0.00049 | 0.000001 |
| 3 | Actin Cytoskeleton Signaling | 86 | 0.00048 | 0.00002 |
| 4 | Axonal Guidance Signaling | 85 | 0.00042 | 0.000009 |
| 5 | Protein Ubiquitination Pathway | 83 | 0.00061 | 0.00002 |
| 6 | Calcium Signaling | 79 | 0.00059 | 0.00002 |
| 7 | Estrogen Receptor Signaling | 79 | 0.00073 | 0.00004 |
| 8 | Molecular Mechanisms of Cancer | 79 | 0.00062 | 0.00002 |
| 9 | Insulin Receptor Signaling | 78 | 0.00118 | 0.00013 |
| 10 | Glucocorticoid Receptor Signaling | 75 | 0.00124 | 0.00034 |
| 11 | Aldosterone Signaling in Epithelial Cells | 74 | 0.0012 | 0.0001 |
| 12 | ERK/MAPK Signaling | 74 | 0.00128 | 0.00016 |
| 13 | Xenobiotic Metabolism Signaling | 74 | 0.00089 | 0.00004 |
| 14 | ILK Signaling | 73 | 0.00106 | 0.00008 |
| 15 | Virus Entry via Endocytic Pathways | 73 | 0.00117 | 0.00007 |
| 16 | Leukocyte Extravasation Signaling | 71 | 0.00151 | 0.00035 |
| 17 | Role of NFAT in Cardiac Hypertrophy | 71 | 0.00121 | 0.00023 |
| 18 | Integrin Signaling | 68 | 0.00082 | 0.00013 |
| 19 | Polyamine Regulation in Colon Cancer | 66 | 0.00108 | 0.00009 |
| 20 | GNRH Signaling | 65 | 0.00166 | 0.00068 |
| 21 | Natural Killer Cell Signaling | 64 | 0.00106 | 0.00015 |
| 22 | CREB Signaling in Neurons | 63 | 0.00164 | 0.00033 |
| 23 | Dopamine-DARPP32 Feedback in cAMP Signaling | 63 | 0.00168 | 0.00042 |
| 24 | Antigen Presentation Pathway | 62 | 0.00078 | 0.00003 |
| 25 | Gustation Pathway | 62 | 0.00161 | 0.00077 |
| 26 | Phospholipase C Signaling | 59 | 0.00213 | 0.00056 |
| 27 | Tight Junction Signaling | 59 | 0.00096 | 0.00018 |
| 28 | Signaling by Rho Family GTPases | 57 | 0.00118 | 0.00048 |
| 29 | Epithelial Adherens Junction Signaling | 56 | 0.00148 | 0.00029 |
| 30 | Hepatic Fibrosis / Hepatic Stellate Cell Activation | 56 | 0.00147 | 0.0003 |
| 31 | B Cell Receptor Signaling | 55 | 0.00126 | 0.00036 |
| 32 | Caveolar-mediated Endocytosis Signaling | 54 | 0.00148 | 0.00073 |
| 33 | G-Protein Coupled Receptor Signaling | 54 | 0.00155 | 0.00053 |
| 34 | Prolactin Signaling | 54 | 0.00175 | 0.00051 |
| 35 | Cdc42 Signaling | 52 | 0.00165 | 0.00083 |
| 36 | PPARÎ±/RXRÎ± Activation | 52 | 0.00133 | 0.00027 |
| 37 | RhoGDI Signaling | 52 | 0.00141 | 0.00037 |
| 38 | Cellular Effects of Sildenafil (Viagra) | 50 | 0.00149 | 0.00037 |
| 39 | Mitochondrial Dysfunction | 50 | 0.00192 | 0.00059 |
| 40 | VDR/RXR Activation | 50 | 0.00133 | 0.00085 |
| 41 | FXR/RXR Activation | 49 | 0.00187 | 0.00077 |
| 42 | Androgen Signaling | 48 | 0.00138 | 0.00052 |
| 43 | Ephrin Receptor Signaling | 48 | 0.00109 | 0.00016 |
| 44 | LXR/RXR Activation | 48 | 0.00147 | 0.00095 |
| 45 | Role of Macrophages, Fibroblasts and Endothelial Cells in Rheumatoid Arthritis | 48 | 0.00154 | 0.00087 |
| 46 | Hereditary Breast Cancer Signaling | 47 | 0.00123 | 0.00041 |
| 47 | Telomerase Signaling | 47 | 0.00143 | 0.00034 |
| 48 | IL-4 Signaling | 45 | 0.00135 | 0.00054 |
| 49 | PI3K/AKT Signaling | 45 | 0.00189 | 0.00063 |
| 50 | Sertoli Cell-Sertoli Cell Junction Signaling | 45 | 0.00142 | 0.0003 |
| 51 | Wnt/Î²-catenin Signaling | 45 | 0.00148 | 0.0006 |
| 52 | Glioma Signaling | 44 | 0.00194 | 0.00102 |
| 53 | Melanocyte Development and Pigmentation Signaling | 44 | 0.00202 | 0.00109 |
| 54 | RAR Activation | 44 | 0.00194 | 0.00048 |
| 55 | Production of Nitric Oxide and Reactive Oxygen Species in Macrophages | 43 | 0.00182 | 0.00044 |
| 56 | Agranulocyte Adhesion and Diapedesis | 42 | 0.00218 | 0.00065 |
| 57 | AMPK Signaling | 42 | 0.00183 | 0.00066 |
| 58 | Corticotropin Releasing Hormone Signaling | 42 | 0.00162 | 0.00039 |
| 59 | HGF Signaling | 41 | 0.0022 | 0.00118 |
| 60 | Oxidative Phosphorylation | 41 | 0.00221 | 0.00106 |
| 61 | phagosome formation | 41 | 0.00142 | 0.00044 |
| 62 | Superpathway of Inositol Phosphate Compounds | 41 | 0.0017 | 0.00063 |
| 63 | Circadian Rhythm Signaling | 40 | 0.00111 | 0.00032 |
| 64 | NGF Signaling | 40 | 0.00119 | 0.00062 |
| 65 | PI3K Signaling in B Lymphocytes | 40 | 0.00222 | 0.00115 |
| 66 | PTEN Signaling | 40 | 0.002 | 0.0008 |
| 67 | RhoA Signaling | 40 | 0.00179 | 0.00089 |
| 68 | Breast Cancer Regulation by Stathmin1 | 39 | 0.00194 | 0.00069 |
| 69 | Chronic Myeloid Leukemia Signaling | 38 | 0.00169 | 0.00074 |
| 70 | Prostate Cancer Signaling | 38 | 0.00183 | 0.00081 |
| 71 | Type II Diabetes Mellitus Signaling | 38 | 0.0017 | 0.00052 |
| 72 | Adipogenesis pathway | 37 | 0.00171 | 0.00104 |
| 73 | Clathrin-mediated Endocytosis Signaling | 37 | 0.00201 | 0.00145 |
| 74 | Synaptic Long Term Depression | 37 | 0.00119 | 0.00031 |
| 75 | Cardiac Hypertrophy Signaling | 36 | 0.00185 | 0.00126 |
| 76 | PKCÎ¸ Signaling in T Lymphocytes | 36 | 0.0026 | 0.00155 |
| 77 | 3-phosphoinositide Biosynthesis | 35 | 0.0023 | 0.00109 |
| 78 | Crosstalk between Dendritic Cells and Natural Killer Cells | 35 | 0.00229 | 0.00131 |
| 79 | FAK Signaling | 35 | 0.00221 | 0.0008 |
| 80 | Macropinocytosis Signaling | 35 | 0.00204 | 0.00101 |
| 81 | mTOR Signaling | 35 | 0.0021 | 0.00122 |
| 82 | Synaptic Long Term Potentiation | 35 | 0.00171 | 0.00093 |
| 83 | Colorectal Cancer Metastasis Signaling | 34 | 0.00209 | 0.00101 |
| 84 | p70S6K Signaling | 34 | 0.00198 | 0.00104 |
| 85 | GPCR-Mediated Nutrient Sensing in Enteroendocrine Cells | 33 | 0.00161 | 0.00077 |
| 86 | Neuregulin Signaling | 33 | 0.00128 | 0.00062 |
| 87 | HIPPO signaling | 32 | 0.00215 | 0.00034 |
| 88 | Rac Signaling | 32 | 0.00207 | 0.00094 |
| 89 | ERK5 Signaling | 31 | 0.0015 | 0.00027 |
| 90 | G Beta Gamma Signaling | 31 | 0.00295 | 0.00141 |
| 91 | Netrin Signaling | 31 | 0.00221 | 0.00103 |
| 92 | Nitric Oxide Signaling in the Cardiovascular System | 31 | 0.00188 | 0.00083 |
| 93 | PPAR Signaling | 31 | 0.00263 | 0.00108 |
| 94 | Regulation of eIF4 and p70S6K Signaling | 31 | 0.00222 | 0.00125 |
| 95 | cAMP-mediated signaling | 30 | 0.00193 | 0.00062 |
| 96 | Fc Epsilon RI Signaling | 30 | 0.00243 | 0.00121 |
| 97 | Non-Small Cell Lung Cancer Signaling | 30 | 0.00228 | 0.00058 |
| 98 | NRF2-mediated Oxidative Stress Response | 30 | 0.00131 | 0.00064 |
| 99 | Regulation of the Epithelial-Mesenchymal Transition Pathway | 30 | 0.00111 | 0.0003 |
| 100 | IL-12 Signaling and Production in Macrophages | 29 | 0.002 | 0.00096 |
| 101 | P2Y Purigenic Receptor Signaling Pathway | 29 | 0.00206 | 0.00111 |
| 102 | Role of BRCA1 in DNA Damage Response | 29 | 0.00157 | 0.00036 |
| 103 | CTLA4 Signaling in Cytotoxic T Lymphocytes | 28 | 0.00207 | 0.00092 |
| 104 | nNOS Signaling in Neurons | 28 | 0.00241 | 0.001 |
| 105 | Ovarian Cancer Signaling | 28 | 0.00164 | 0.00095 |
| 106 | Cardiac Î²-adrenergic Signaling | 27 | 0.00297 | 0.00295 |
| 107 | eNOS Signaling | 27 | 0.00219 | 0.00063 |
| 108 | Factors Promoting Cardiogenesis in Vertebrates | 27 | 0.0023 | 0.00114 |
| 109 | LPS/IL-1 Mediated Inhibition of RXR Function | 27 | 0.00125 | 0.00051 |
| 110 | Mitotic Roles of Polo-Like Kinase | 27 | 0.00251 | 0.00168 |
| 111 | PDGF Signaling | 27 | 0.00195 | 0.00123 |
| 112 | 3-phosphoinositide Degradation | 26 | 0.00166 | 0.00126 |
| 113 | D-myo-inositol-5-phosphate Metabolism | 26 | 0.00115 | 0.00049 |
| 114 | Germ Cell-Sertoli Cell Junction Signaling | 26 | 0.00193 | 0.00122 |
| 115 | Neuropathic Pain Signaling In Dorsal Horn Neurons | 26 | 0.00187 | 0.00095 |
| 116 | nNOS Signaling in Skeletal Muscle Cells | 26 | 0.00199 | 0.0013 |
| 117 | TR/RXR Activation | 26 | 0.00204 | 0.00085 |
| 118 | Acute Phase Response Signaling | 25 | 0.00222 | 0.00159 |
| 119 | D-myo-inositol (1,4,5,6)-Tetrakisphosphate Biosynthesis | 25 | 0.00114 | 0.00086 |
| 120 | Neuroprotective Role of THOP1 in Alzheimer's Disease | 25 | 0.00268 | 0.00116 |
| 121 | p38 MAPK Signaling | 25 | 0.00215 | 0.00091 |
| 122 | Reelin Signaling in Neurons | 25 | 0.00137 | 0.00071 |
| 123 | Role of Osteoblasts, Osteoclasts and Chondrocytes in Rheumatoid Arthritis | 25 | 0.0022 | 0.00106 |
| 124 | ATM Signaling | 24 | 0.00255 | 0.00099 |
| 125 | Bladder Cancer Signaling | 24 | 0.00218 | 0.00176 |
| 126 | D-myo-inositol (3,4,5,6)-tetrakisphosphate Biosynthesis | 24 | 0.00101 | 0.00072 |
| 127 | FcÎ³ Receptor-mediated Phagocytosis in Macrophages and Monocytes | 24 | 0.00259 | 0.00094 |
| 128 | Aryl Hydrocarbon Receptor Signaling | 23 | 0.00155 | 0.00086 |
| 129 | Cell Cycle: G1/S Checkpoint Regulation | 23 | 0.00267 | 0.0012 |
| 130 | Agrin Interactions at Neuromuscular Junction | 22 | 0.00133 | 0.0009 |
| 131 | CDK5 Signaling | 22 | 0.00202 | 0.00095 |
| 132 | FLT3 Signaling in Hematopoietic Progenitor Cells | 22 | 0.0017 | 0.00061 |
| 133 | Glycolysis I | 22 | 0.00254 | 0.0016 |
| 134 | p53 Signaling | 22 | 0.002 | 0.00083 |
| 135 | TGF-Î² Signaling | 22 | 0.00219 | 0.00093 |
| 136 | Allograft Rejection Signaling | 21 | 0.00402 | 0.00393 |
| 137 | GABA Receptor Signaling | 21 | 0.00321 | 0.00234 |
| 138 | Pancreatic Adenocarcinoma Signaling | 21 | 0.00326 | 0.00188 |
| 139 | Renin-Angiotensin Signaling | 21 | 0.00224 | 0.00115 |
| 140 | Small Cell Lung Cancer Signaling | 21 | 0.00191 | 0.00071 |
| 141 | Sperm Motility | 21 | 0.00323 | 0.00212 |
| 142 | ErbB4 Signaling | 20 | 0.00175 | 0.00065 |
| 143 | IGF-1 Signaling | 20 | 0.00271 | 0.00206 |
| 144 | Maturity Onset Diabetes of Young (MODY) Signaling | 20 | 0.003 | 0.00121 |
| 145 | Melatonin Degradation I | 20 | 0.0019 | 0.00117 |
| 146 | Nicotine Degradation III | 20 | 0.00212 | 0.00116 |
| 147 | OX40 Signaling Pathway | 20 | 0.00361 | 0.00287 |
| 148 | Paxillin Signaling | 20 | 0.00221 | 0.00137 |
