# Supplementary material for: Whole exome sequencing reveals HSPA1L as a genetic risk factor for spontaneous preterm birth
Source: PLoS Genet. 2018 Jul 12;14(7):e1007394. doi: 10.1371/journal.pgen.1007394 (PMC6042692; doi:10.1371/journal.pgen.1007394)
Supplement: S4 Table — (DOCX) [file pgen.1007394.s008.docx]

**S4 Table. Comparison of variant filtering results from different analysis software used for Finnish families with multiple affected individuals (n=5).**

| **Software** | **Average number of variants^4^ (range)** | **Average number of genes (range)** | **No. of common genes for ≥2 families** | **No. of unique genes for single family** |
| --- | --- | --- | --- | --- |
| **Ingenuity Variant Analysis^1^** | 444 (278–691) | 243 (173–381) | 168 | 763 |
| **Varseq^2^** | 136 (61−244) | 120 (52−215) | 44 | 497 |
| **Warehouse^3^** | 282 (231–424) | 253 (216–351) | 104 | 658 |

^1^Included only rare variants (MAF <1% in the 1000 Genomes Project, ExAC or in European American population in NHLBI ESP exomes) with likely functional effect (predicted pathogenic or likely pathogenic by SIFT or PolyPhen-2, listed in Human Gene Mutation Database, or associated with gain or loss of function of a gene). ^2^Included only rare (MAF <1% in the general Finnish population) and missense or loss-of-function variants. ^3^Included only rare (MAF <1% in the Children’s Mercy Hospital Variant Warehouse database) and predicted pathogenic variants. ^4^Rare heterozygous variants that were shared by 2−3 affected individuals per family and that passed the annotation and prioritizing filters within the software.
