# Supplementary material for: Whole exome sequencing reveals HSPA1L as a genetic risk factor for spontaneous preterm birth
Source: PLoS Genet. 2018 Jul 12;14(7):e1007394. doi: 10.1371/journal.pgen.1007394 (PMC6042692; doi:10.1371/journal.pgen.1007394)
Supplement: S5 Table — (DOCX) [file pgen.1007394.s009.docx]

**S5 Table. Functional categories of rare variants passing the annotation and prioritizing steps of multiple software tools.**

| **Population set** | **Variant Category** |  | **Total no. of Different variants** | **No. of Variants** | **No. of Genes** | **No. of Variants with *in silico* Pathogenicity Prediction as Damaging^1-5^** | | | | |
| --- | --- | --- | --- | --- | --- | --- | --- | --- | --- | --- |
|  |  |  |  |  |  | **SIFT^1^** | **PolyPhen2 HVAR^2^** | **Mutation Taster^3^** | **Mutation Assessor^4^** | **FATHMM^5^** |
| Discovery  (n=5 families) | Loss of Function | Start loss | 844 | 2 | 2 | 1 | 2 | 1 | . | . |
|  |  | Stop gain |  | 24 | 24 | . | . | 8 | . | . |
|  |  | Frameshift |  | 27 | 20 | . | . | . | . | . |
|  | Moderate | Missense |  | 714 | 652 | 228 | 316 | 245 | 120 | 69 |
|  |  | In-frame indel |  | 19 | 19 | . | . | . | . | . |
|  | Other | Splice site^6^ |  | 43 | 42 | . | . | 2 | . | . |
|  |  | Other |  | 15 | 13 | . | . | 1 | . | . |
|  |  |  |  |  |  |  |  |  |  |  |
| Replication  (n=93 families) | Loss of Function | Start loss | 8431 | 18 | 18 | 15 | 17 | 13 | . | 2 |
|  |  | Stop loss |  | 12 | 12 | . | . | 5 | . | . |
|  |  | Stop gain |  | 218 | 211 | 2 | 2 | 198 | 1 | . |
|  |  | Frameshift |  | 232 | 225 | . | . | . | . | . |
|  | Moderate | Missense |  | 6977 | 4970 | 3227 | 4476 | 5506 | 2848 | 1266 |
|  |  | In-frame indel |  | 832 | 661 | 1 | 1 | 1 | 1 | . |
|  | Other | Splice acceptor |  | 49 | 49 | . | . | . | . | 32 |
|  |  | Splice donor |  | 76 | 76 | . | . | 55 | . | . |
|  |  | 5’ UTR |  | 17 | 17 | . | . | 5 | . | . |

^1^Damaging, ^2^Probably damaging, possibly damaging, ^3^Disease causing, disease causing automatic, ^4^Predicted functional (medium), predicted functional (high), ^5^Damaging. ^6^Splice site change/loss; splice acceptor, splice donor and splice region variants. Most variants overlap several *in silico* pathogenicity predictions (not specified).
