# Supplementary material for: Whole exome sequencing reveals HSPA1L as a genetic risk factor for spontaneous preterm birth
Source: PLoS Genet. 2018 Jul 12;14(7):e1007394. doi: 10.1371/journal.pgen.1007394 (PMC6042692; doi:10.1371/journal.pgen.1007394)
Supplement: S6 Table — (DOCX) [file pgen.1007394.s010.docx]

**S6 Table. Functional categories of rare variants common for both Discovery and Replication populations.**

| **Category** | **Translation Impact** | **No. of shared variant** | **No. of Genes** | **Example genes** |
| --- | --- | --- | --- | --- |
| 1. Loss of Function | Stop gain | 2 | 2 | *FAM173B*, *ABCC12* |
| 2. Moderate | Missense [damaging]^1^ | 31 | 31 | *ABCC2*, *ADAMTSL5*, *ASCC2*, *C1orf94*, *CADPS2*;*RNF133*, *CALCOCO2*, *CHAT*, *COL13A1*, *DHTKD1*, *DSG2*, *ENPP2*, *GADD45GIP1*, *GTPBP10*, *IGSF1*, *KATNAL1*, *KIF13A*, *KIF26A*, *KPNA2*, *LYST*, *MLST8*;*BRICD5*, *MYH9*, *NWD1*, *OR2F1*, *PHKB*, *PIGW*, *PRKCE*, *SEC31B*, *SRP68*, *TAS2R41*, *TRPM3*, *ZKSCAN5* |
|  | Missense [tolerated and other]^2^ | 39 | 39 | *AAR2*, *B4GALT4*, *C10orf129*, *C13orf45*, *C1orf27*, *CCDC144NL*, *CCDC168*, *CTBP2*, *CYSLTR2*, *DCDC5*, *DUSP26*, *EP400*, *FAM170A*, *FREM3*, *FSIP2*, *HELQ*, *HS6ST2*, *HTRA4*, *IGHMBP2*, *KCNC2*, *LOC100132146*, *MBD1*, *MEFV*, *MYBPC2*, *NEK7*, *OTOG*, *PHLDB1*, *PKHD1*, *PNISR*, *PTPN14*, *RABL6*, *SMIM3*, *SPATA18*, *SYNRG*, *TCHH*, *TTN*, *TUBGCP5*, *ZNF135*, *ZNF330* |

^1^Most (≥3 of 5) *in silico* prediction tools (SIFT, PolyPhen2, Mutation Taster, Mutation Assessor or FATHMM) predicted as damaging. ^2^Most *in silico* predictions predicted as tolerated or no prediction was available.
