# Supplementary material for: Whole exome sequencing reveals HSPA1L as a genetic risk factor for spontaneous preterm birth
Source: PLoS Genet. 2018 Jul 12;14(7):e1007394. doi: 10.1371/journal.pgen.1007394 (PMC6042692; doi:10.1371/journal.pgen.1007394)
Supplement: S7 Table — (DOCX) [file pgen.1007394.s011.docx]

**S7 Table. Relative HSPA1L and GR protein levels in cytosolic and nuclear extracts; supplementing the Fig 3.**

| **HSPA1L cytosolic** | | | **GR cytosolic** | | |
| --- | --- | --- | --- | --- | --- |
| Control | WT | Ala268Thr | Control | WT | Ala268Thr |
| 0.762 | 0.869 | 0.357 | 0.775 | 0.952 | 0.517 |
| 0.834 | 0.917 | 0.396 | 0.825 | 1.007 | 0.652 |
| 0.871 | 1.058 | 0.596 | 0.894 | 1.107 | 0.903 |
| 0.920 | 1.078 | 0.697 | 0.903 | 1.183 | 0.916 |
| 0.926 | 1.093 | 0.780 | 0.978 | 1.202 | 0.946 |
| 1.049 | 1.170 | 0.970 | 1.099 | 1.314 | 1.108 |
| 1.121 | 1.280 | 1.263 | 1.137 | 1.598 | 1.245 |
| 1.255 | 1.975 | 1.436 | 1.158 | 1.641 | 1.248 |
| 1.261 | 2.005 | 1.546 | 1.232 | 1.778 | 1.402 |
| **HSPA1L nuclear** | | | **GR nuclear** | | |
| Control | WT | Ala268Thr | Control | WT | Ala268Thr |
| 0.308 | 0.891 | 0.983 | 0.447 | 0.727 | 0.732 |
| 0.444 | 1.041 | 0.990 | 0.783 | 0.761 | 0.750 |
| 0.863 | 1.077 | 1.088 | 0.807 | 0.791 | 0.843 |
| 0.937 | 1.106 | 1.107 | 0.836 | 0.802 | 0.888 |
| 0.977 | 1.301 | 1.281 | 0.855 | 1.008 | 0.911 |
| 1.221 | 1.607 | 1.288 | 1.080 | 1.194 | 1.049 |
| 1.578 | 1.923 | 1.855 | 1.500 | 1.872 | 1.655 |
| 1.672 | 2.085 | 2.059 | 1.691 | 1.958 | 1.698 |
|  | 2.102 | 2.202 |  | 2.008 | 1.742 |
